# Prey availability and foraging activity by tundra‐nesting sea ducks: Strong preference for specific wetland types

**DOI:** 10.1002/ece3.10375

**Published:** 2023-09-20

**Authors:** Micah W. C. Miller, James R. Lovvorn, Nathan R. Graff, Neesha C. Stellrecht, Steven P. Plesh

**Affiliations:** ^1^ Department of Zoology and Center for Ecology Southern Illinois University Carbondale Illinois USA; ^2^ U.S. Fish and Wildlife Service, Northern Alaska Fish and Wildlife Field Office Fairbanks Alaska USA

**Keywords:** Arctic birds, climate change, eider, long‐tailed duck, thawing tundra, tundra wetlands, wetland invertebrates

## Abstract

Wetlands in Arctic tundra support abundant breeding waterbirds. Wetland types differing in area, depth, vegetation, and invertebrate biomass density may vary in importance to birds, and in vulnerability to climate change. We studied availability and use of different wetland types by prelaying females of four species of sea ducks (Mergini) breeding on the Arctic Coastal Plain of Alaska, USA: long‐tailed ducks (*Clangula hyemalis*) and Steller's (*Polysticta stelleri*), spectacled (*Somateria fischeri*), and king eiders (*Somateria spectabilis*). All four species preferred shallow vegetated wetlands versus deeper lakes. The ducks spent almost all their active time feeding, but their occurrence in different wetland types was not affected by the relative biomass density of known prey or of all invertebrates that we sampled combined. Sea ducks strongly preferred wetlands dominated by emergent and submersed *Arctophila fulva* over those dominated by the sedge *Carex aquatilis*, despite the much greater number, total area, and invertebrate biomass density of *Carex* wetlands. The hens depend heavily on local invertebrate prey for protein to produce eggs; thus, their preference for *Arctophila* wetlands likely reflects greater accessibility of prey in the near‐surface canopy and detritus of *Arctophila*. Such shallow wetlands decreased substantially in number (−17%) and area (−30%) over 62 years before 2013 and appear highly susceptible to further declines with climate warming. Impacts on sea ducks of climate‐driven changes in availability of important wetland types will depend on their adaptability in exploiting alternative wetlands.

## Graphical abstract

Sea ducks rely on food from tundra wetlands during the breeding season. We compared patterns of wetland use by female sea ducks of four species to the availability of different wetland types wetlands and prey, as well as behaviors of sea ducks within these wetlands. Wetlands of most apparent value comprise a small fraction of wetland area, and have moderate invertebrate prey biomass densities, but are highly selected for by female sea ducks.

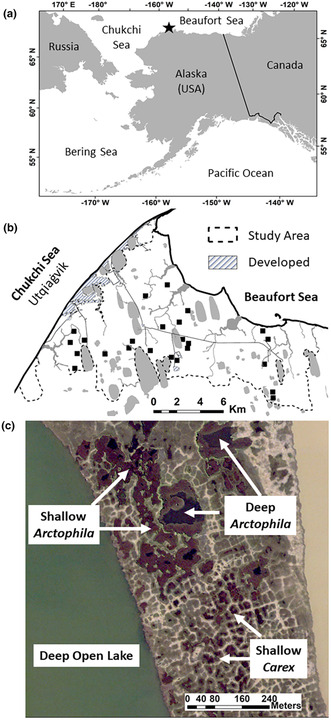

## 1 INTRODUCTION

Across the Arctic, tundra wetlands provide critical foraging habitat for breeding migratory birds, including waterfowl, jaegers, gulls, terns, and shorebirds. However, in recent decades, populations of many of these species have declined substantially (Smith et al., 2020; Wauchope et al., 2017). Reasons for these declines are complex but are likely related to disproportionate climate change by which the Arctic is warming two to four times faster than the rest of the globe (Previdi et al., 2021; Rantanen et al., 2022; Wauchope et al., 2017).

In particular, sea ducks (Mergini) that nest in the North American Arctic have shown major declines from historic levels (Bowman et al., 2015; Suydam et al., 2000), with some evidence for recent increases especially on the subarctic Yukon‐Kuskokwim Delta (Amundson et al., 2019; Bowman et al., 2015; Dunham et al., 2021). Because sea ducks nest on land but winter at sea, identifying when and where during the annual cycle population limitations occur is a major challenge. Cross‐seasonal effects, such as nutrient limitation, may have population‐level impacts on sea ducks (Alisauskas & Devink, 2015). On the Arctic Coastal Plain of Alaska, recent studies indicate that sea ducks arriving after a winter at sea rely heavily on invertebrate prey in tundra wetlands to fuel the costs of egg‐laying and incubation (Miller et al., 2022). However, the biomass of different invertebrate foods varies among wetland types (Plesh et al., 2023), and climate warming is substantially altering the availability of different wetland types on the landscape (Andresen & Lougheed, 2015). To project how these changes in wetland availability might affect future habitat for sea ducks, it is important to know how different species are distributed among wetland types; how they use wetlands for foraging, resting, courtship, and other behaviors; and what options may exist for adapting to change (Bergman et al., 1977; Kondratiev & Zadorina, 1992; Kondratyev, 1999; Oppel et al., 2011; Solovieva, 2005). Such information is especially valuable during the critical period of nest establishment and egg‐laying.

On the Alaskan Arctic Coastal Plain, the number and surface area of individual ponds are declining through both increased production of emergent vegetation and draining of wetlands via permafrost thaw (Andresen & Lougheed, 2015). Indeed, for tundra wetlands near Utqiaġvik, Alaska, roughly 30% of the total pond area, and 17% of total pond number, were lost between 1948 and 2013, with higher rates of shrinkage and loss occurring in shallower wetlands (Andresen & Lougheed, 2015). Over that same period, mean summer temperature (Andresen & Lougheed, 2015) and snow‐free season length (Cox et al., 2017) both increased, while the onset of spring progressed by 16 days from 1992 to 2016 (Amundson et al., 2019).

We studied four species of sea ducks of international conservation concern (BirdLife International, 2022a, 2022b, 2022c, 2022d; USFWS, 2019, 2021) which breed sympatrically in northern Alaska: long‐tailed ducks (*Clangula hyemalis*, LTDU) and Steller's (*Polysticta stelleri*, STEI), spectacled (*Somateria fischeri*; SPEI), and king (*S. spectabilis*, KIEI) eiders. In northern Alaska, foods in tundra wetlands comprised most proteins used for egg production, and at least half of proteins used by females to sustain themselves throughout incubation, with the remainder coming from stored reserves derived from marine foods (Miller et al., 2022). In fact, females of some species (Steller's and common eiders *S. mollissima*) are known to forego breeding locally in some years, a decision that for common eiders was based in part on female body condition before nest initiation (Coulson, 2010; Quakenbush et al., 2004). Foods in local wetlands may offset effects of migration and low body condition on breeding, allowing these birds to gain sufficient lipid and protein stores to produce eggs and sustain themselves through incubation (e.g., Bond et al., 2007; Miller et al., 2022). Thus, availability of prey in different wetland types, and the occurrence of those types, could impact the ability of females to acquire nutrients for reproduction. Sea ducks often feed mostly on benthic prey, and in marine habitats they typically dive to forage. However, in freshwater habitats (as in the tundra), they exhibit a range of foraging behaviors from diving to surface feeding depending on water depth (e.g., Solovieva, 2005 for Steller's eiders).

Given ongoing and probable future changes in the availability of different wetland types that support different biomasses of invertebrates (Plesh et al., 2023), we investigated the occurrence of females of four sea duck species relative to wetland type and invertebrate prey biomass, and patterns of behavior (especially foraging behavior) of the different duck species. We asked the following questions:

1. What are the patterns of occurrence of female sea ducks on different wetland types?
2. How are patterns of female sea duck occurrence associated with density of invertebrate prey biomass?
3. How do females of different sea duck species allocate their time among behaviors, and especially among foraging modes?

We then interpret these results in terms of the birds' selection of different wetland types, possible reasons for those preferences, and how climate change is likely to alter future habitat conditions for these species.

## 2 MATERIALS AND METHODS

### 2.1 Study area

**FIGURE 1:**
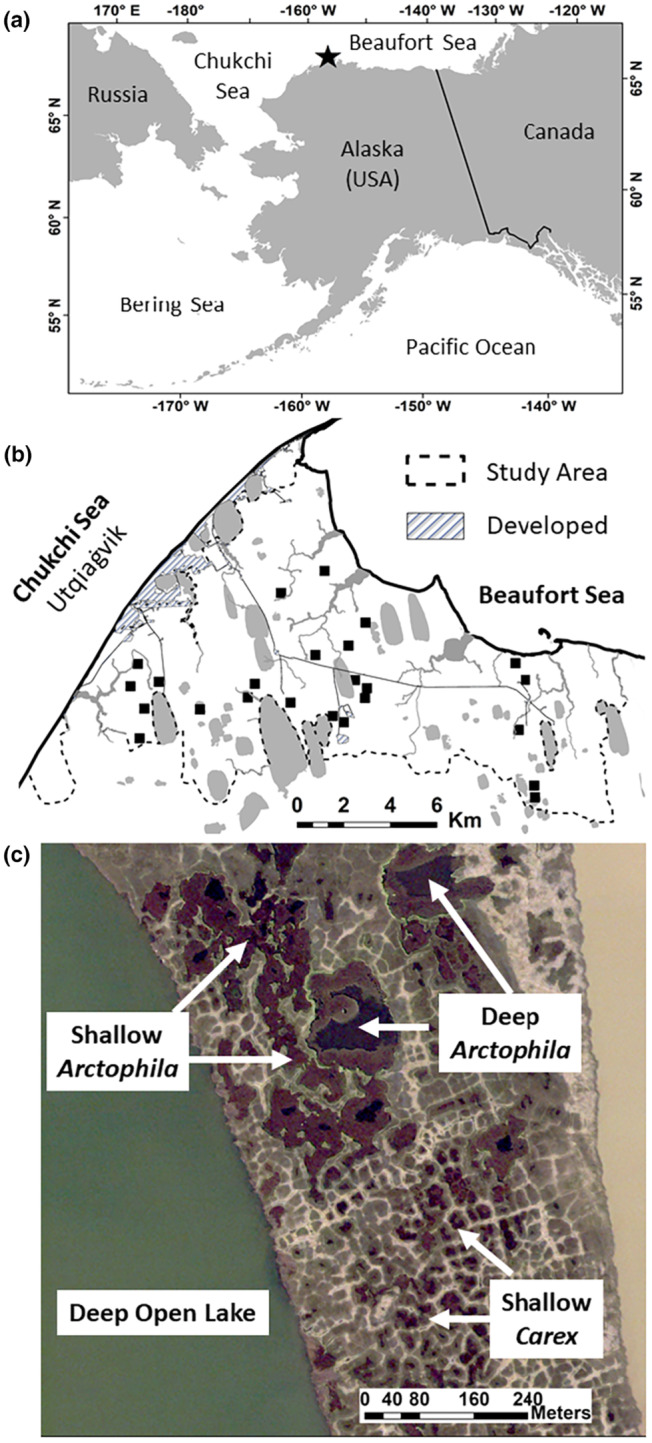
(a) Study location (black star) near Utqiaġvik, Alaska. (b) Study area showing locations of ground‐surveyed 400 × 400 m randomly‐located wetland plots (black rectangles) relative to human developments and major water bodies (gray). (c) Examples of adjacent wetlands of variable types in a landscape of polygonal tundra and thaw lake basins. Note extensive emergent *Arctophila* in some wetlands.

Our study area near Utqiaġvik (formerly Barrow), Alaska, encompasses ~175 km^2^ of coastal tundra within 5 km of the road system (Graff, 2021; Figure 1a,b). The Ukpeaġvik Iñupiat Corporation permitted foot‐based tundra access, with road and beach access by ATV or 4WD vehicle. Our study area is mostly wet tundra interspersed with wetlands ranging from small, shallow basins to large, deep lakes and flowing streams.

This study includes data collected from May–July 1999–2019, with most work completed in 2017–2019. Snowmelt occurs over a period of a few weeks from late May into June. Wetlands are generally full just after snowmelt, and progressively become shallower through the summer owing to evapotranspiration. The timing of melt shifted ~16 days earlier from 1992 to 2016 (Figure A2.2 in Amundson et al., 2019). Large ephemeral basin marshes (Bergman et al., 1977) fill with meltwater before draining to form discrete wetlands later in the season. High concentrations of birds use these basins before dispersing to breeding sites.

### 2.2 Conservation status of study species

We focused our surveys on four focal sea duck species that breed within our study area: long‐tailed ducks and Steller's, spectacled, and king eiders. Population trends of these species in northern Alaska vary spatiotemporally but are largely stable to slightly declining (Amundson et al., 2019); however, populations are well below historic levels (BirdLife International, 2022a, 2022b, 2022c, 2022d). Steller's and spectacled eiders are currently listed as Threatened under the U.S. Endangered Species Act (U.S. Fish and Wildlife Service, 2019, 2021), with Steller's eiders breeding mainly near our study area in Utqiaġvik. While population data are largely lacking within our study area, long‐tailed ducks are by far the most abundant sea duck.

### 2.3 Occurrence of sea ducks in wetlands

**FIGURE A1:**
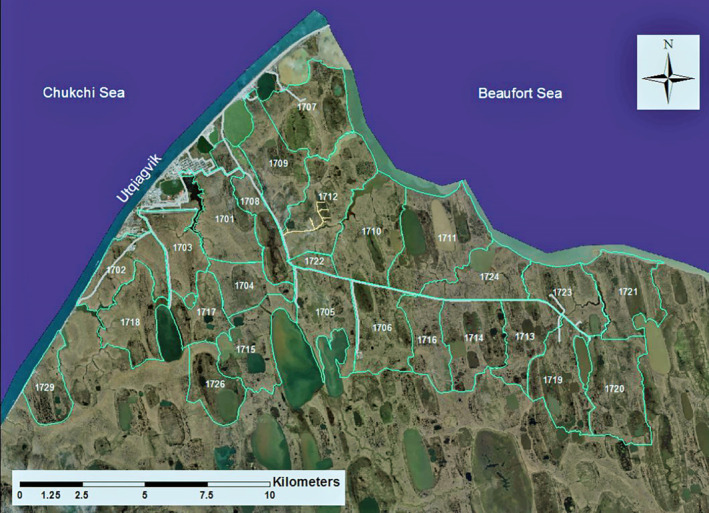
2017 ground‐based survey subunits. The 2017 survey was completed over 12 days in mid‐late June, surveying from west to east over this period.

We integrated multiple occurrence datasets for this study, with most occurrence data from ground‐based occurrence surveys and lesser amounts from incidental observations and from focal behavioral sampling (Table 1). From mid‐June to early July 1999–2019, ground‐based occurrence surveys were completed throughout our ~175 km^2^ study area (Figures 1 and A1) to document the locations and numbers of Steller's and spectacled eiders each year. Our survey objective was to achieve equal spatial coverage across the landscape, rather than sampling equal proportions of different wetland types (Figure 1c) which varied greatly in number and area. Briefly, ~28 (range 21–30) discrete survey units (Figure A1, mean ± SD = 6.67 ± 2.1 km^2^) were sampled once each year by groups of ~5 (2–13) observers walking parallel throughout each subunit (Figure A1); 1–3 units were surveyed each day. These surveys were designed to provide nearly complete coverage of our ~175‐km^2^ study area, so they included almost all wetlands. Surveys were initiated when snowmelt was nearly completed and birds had dispersed from seasonally flooded staging wetlands into smaller wetlands (presumably near breeding sites), so survey dates varied across years with timing of snowmelt but followed similar phenology; in all but 2 years surveys were completed before 30 June (Table A1). The location, habitat type, and occupancy code were recorded for each individual female (Appendix 1). Occupancy categories ranged from flying over (and thus not occurring within a specific habitat type) to confirmed nesting. Surveys focused exclusively on Steller's and spectacled eiders from 1999 to 2016. King eiders were added in 2017–2019. Detailed information on annual survey methods and effort is in Appendix 1.

**TABLE 1:** Filtered sea duck occurrence data, 1999‐2019. For filtering methods, see Appendix 2.

Species	Occurrence surveys (1999–2019)	Behavior observations (2017–2019)	Incidental (2017–2019)	Total occurrences (All years)
Long‐tailed duck	Not recorded	621	803	1424
Steller's eider	814	329	182	1325
Spectacled eider	543	88	63	694
King eider	33 (2017–2019 only)	153	170	353

In addition to these ground‐based surveys, we also recorded occurrences of female sea ducks during behavioral observations (see the section below) and incidentally while completing other work during the prebreeding period. All occurrence data on long‐tailed ducks derive from these two alternate datasets. Details on data integration and filtering for the compiled occurrence dataset are in Appendix 2 and Table A2.

We did not account for variable detection of female sea ducks among wetland types or years. Our study occurred early in the breeding season, during or immediately after snowmelt. Emergent plants and peripheral vegetation along shorelines senesced each winter and were at annual minima. Vegetation that was present was typically lodged, flooded, and provided little to no concealment for birds in any wetland type. During behavior observations (see section below) birds moved out of sight on some occasions. Such rare cases occurred almost entirely during observations at low angles and moderate distances, where intervening snow and ice limited our ability to detect individual birds. Thus, our bird observations are potentially biased during the earliest samples in any year, but biases are likely consistent among duck species, years, and wetland types.

We also avoided spatial analyses for our occurrence dataset, as much of the data was collected with imprecise GPS (horizontal point precision ±30 m; M. W. C. Miller, unpublished data) or locations estimated spatially by observers (e.g., observer location was known, but the distance and direction from observer to the sea duck was estimated but not consistently recorded). To avoid these potentially substantial biases for spatial analyses, such as occupancy modeling, we instead used the summarized proportions of sea duck occurrences in each wetland type at a broad scale but acknowledge that improving the precision of occurrence locations would facilitate such analyses.

### 2.4 Wetland surveys and delineation

To determine the relative area of wetlands, we used a combination of ground‐based wetland delineation and remote sensing datasets to delineate wetland boundaries. Briefly, we ground‐surveyed 400 × 400 m randomly‐located plots (*n* = 24; Figure 1b) to determine the relative frequency of each wetland type on the landscape, classifying 1652 wetlands during 2017 and 2018 (Table 2). Wetlands were classified by their dominant vegetation (either the emergent grass *Arctophila fulva* or sedge *Carex aquatilis*) and water depth (following Bergman et al., 1977; see also Table A3). Ponds <40 cm deep were considered shallow, and those with depth > 40 cm were considered deep; many deep wetlands were nearly 1 m deep. This classification also included Deep Open Lakes (depth > 1 m, surface area > 1 ha) and Streams. Deep Open Lakes were mostly >2 m deep, with some up to 8 m deep, but all of our invertebrate sampling was in littoral zones <1 m deep that were accessible by wading. We pooled occurrence data for sea ducks observed in Deep Open Lakes and Deep *Carex* ponds (referred to hereafter as Deep Lakes), as these were not differentiated in earlier years of our two‐decade survey dataset.

**TABLE 2:** Mean surface area per wetland, and percentages of total wetland area, for different wetland types, were calculated across 24 plots of 1600 m^2^ each (total of 0.0384 km^2^, see Appendix 1).

Wetland type	Surface area per wetland (ha)	% of total wetland area
	Mean ± SD (*n*)	
Shallow *Arctophila*	0.09 ± 0.21 (117) a	1.3
Deep *Arctophila*	0.73 ± 2.97 (62) cd	4.8
Shallow *Carex*	0.05 ± 0.63 (749) b	5.2
Deep Lakes	5.67 ± 21.12 (157) d	78.6
Streams	4.88 ± 13.18 (24) c	10.1

*Note*: Not all wetlands documented in plots were used to estimate surface areas (Appendix 2). The percentage of total wetland area was across all plots, rather than means among plots (see Section 2.4). Shared lower‐case letters indicate no difference in mean surface area among wetland types (Kruskal–Wallis *H* test, *p* > .05).

**FIGURE A2:**
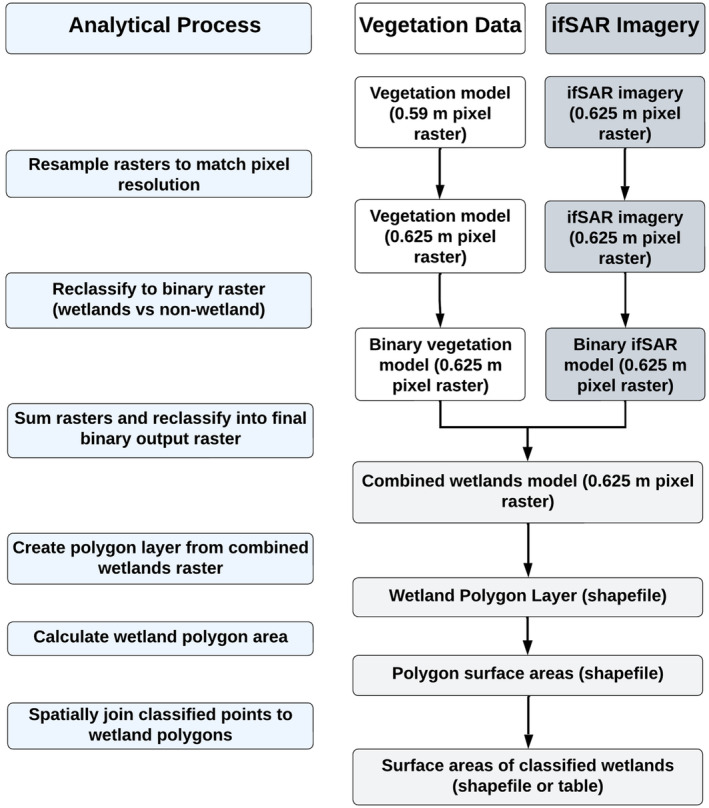
Remote‐sensing workflow.

To delineate wetland perimeters, we used a vegetation model derived from WorldView orthorectified satellite imagery (Andresen et al., 2017; Lara et al., 2016) and inferometric serial aperture radar (ifSAR; U.S. Geologic Survey, 2021), from which we calculated surface area of individual wetlands (detailed methods in Appendix 2, Figure A2). We multiplied the number of each wetland type (from ground surveys) by the mean surface area for that wetland type (from remote sensing) to estimate the total wetland surface area for each type. As our approach was broad and we were interested in relative wetland surface area for sea ducks, we used locations of wetlands, and their respective area, pooled across all plots to get a metric of landscape availability, rather than variance among ground‐based wetland plots. This aggregate approach was more representative of relative variability at a landscape scale than assessment of mean wetland area among wetland plots.

We were not able to delineate Streams with remote‐sensing datasets, as tundra streams are actually a series of interconnected pools (beads) with ephemeral connectivity. Snowmelt can cause streams to swell well beyond the perimeters of the individual beads, yet we were unable to consistently measure the area included given spatiotemporal variation in snowmelt phenology. Instead, we manually delineated stream boundaries by assuming that vegetation that is seasonally inundated is distinct from adjacent vegetation in the imagery, so that seasonally flooded vegetation adjacent to beads was inundated each spring and represented available habitat for pre‐laying and laying sea ducks.

### 2.5 Invertebrate sampling and biomass estimation

**FIGURE A3:**
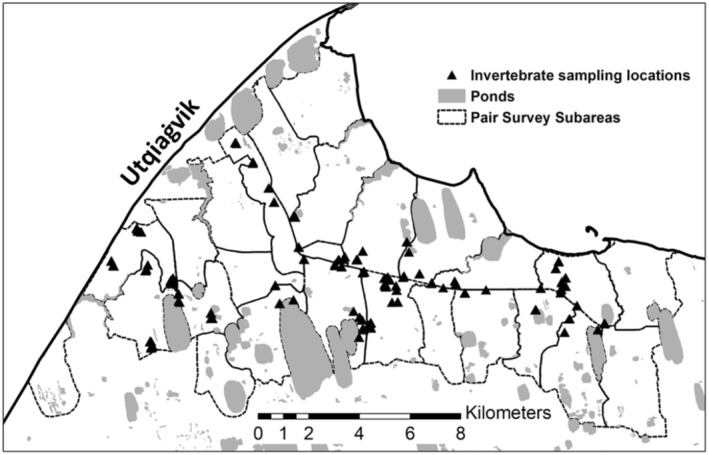
Invertebrate sampling locations, 2017–2018. Black triangles indicate wetlands sampled for invertebrates and estimate biomass density. Pair survey subareas are delineated in dashed lines.

Invertebrate sampling methods are described in detail by Miller et al. (2022) and Plesh et al. (2023). In June and July of 2017 and 2018, we sampled each wetland with benthic cores (*n* = 4) and net sweeps through emergent vegetation (*n* = 2) (Figure A3, Table A6). We treated cores and sweeps separately for all comparisons. Samples were sifted with water from the sampled wetland through a 0.5‐mm sieve and then stored frozen; samples were later thawed, sorted by taxon, and counted to estimate the abundance of each taxon in cores and sweeps separately. Most invertebrates in cores were in the top centimeter of sediments, so volume correction for cores was not necessary.

Samples were frozen for use in stable isotope studies (Miller et al., 2022; Plesh et al., 2023), during which total C content was measured for aggregated cores and aggregated sweeps within each individually sampled wetland. We divided the C content of these aggregate samples by the number of individuals that comprised each sample and applied resulting per‐individual C values to field samples of invertebrate abundance to estimate C biomass density for each taxon based on cores (g C/m^2^) and sweeps (g C/m^3^) separately within each wetland. Our estimates of C biomass density were representative of each sample type and wetland individually, rather than an aggregate across wetlands of each type. We then averaged the means from different wetlands to yield a mean C biomass density for each wetland type.

Total invertebrate biomass within wetlands is proportional to wetland area; large wetlands with low prey densities may have seemingly large prey biomass yet little value to foraging birds due to low energies densities per area and high costs to locate foods (Lovvorn et al., 2013). To avoid confounding wetland area with total prey biomass, we opted instead to use invertebrate biomass density, defined as mg C per unit area or volume. Carbon is a widely used metric of energy transfer in trophic studies (Plesh et al., 2023), and the density of C may be a major factor in profitability to foraging birds.

Our sampling was inadequate to quantify the biomass density of oligochaetes, which stable isotope studies later revealed could be important prey for sea ducks (14%–52% of the diet for the four species studied here, Miller et al., 2022). Oligochaetes vary widely in their relative size and are often considered meiofauna; thus, they are not sampled well by a 0.5‐mm sieve which we used as the accepted standard for macroinvertebrate sampling (Higgins & Thiel, 1988; Hummon, 1981; Nalepa & Robertson, 1981). Moreover, despite observing oligochaetes in both sweeps and cores in the field, very few were recovered after samples frozen for stable isotope analyses were thawed, consistent with a reported sampling bias after storage that affects this taxon (Jónasson, 1955). Because we did not anticipate stable isotope results that later indicated the importance of meiofaunal oligochaetes in sea duck diets, we did not develop a different method for sampling oligochaetes effectively during our fieldwork and could not consider them here.

### 2.6 Behavior observations

We analyzed behaviors of all four sea duck species in 2017 and 2018, and 2019 only for spectacled and king eiders to augment low sample sizes in the previous years. Observations were conducted in areas accessible by road from late May until late June, when females were acquiring nutrients from tundra wetlands for reproduction (Miller et al., 2022). As snowmelt progressed each year, a wider range of wetlands were accessible for behavioral observations. During this period, females were either in small groups or with their mates. At each sampling location where sea ducks were present, we arbitrarily selected an initial focal female to observe and watched each female sequentially until all present had been observed before moving to a new location. We recorded behaviors (including any diving) continuously throughout 5‐min focal‐individual periods from inside a vehicle using 20 to 60× spotting scopes and digital audio recorders.

Behaviors were assigned to five major categories (foraging, loafing, locomotion, reproduction, and out‐of‐view), with more specific behaviors fitting in each broad category (Table A4). We recorded behaviors at 20‐s intervals yielding 16 observations per 5‐min sampling period (Lovvorn, 1989). In some cases, groups of females exhibited similar behaviors. When possible, each female was observed separately, but some females were observed collectively, e.g., when sleeping in tight groups. If the specific type of behavior was uncertain, but the general category was known (e.g., foraging), then the more general category was used instead of precise behaviors (Table A4). Because daylight is not limited at this latitude and season, sampling was distributed throughout the 24‐h cycle. As females were not individually marked, we waited for ≥1 h between observation periods of suspected individual females. In many cases, focal females were 200–500 m from the observation vehicle, and thus were not always clearly visible (out‐of‐view). If a female was not visible for >2 min in any 5‐min focal period, that sampling period was excluded from activity budget calculations. We calculated the proportion of observations during each 5‐min period that were allocated to each behavior.

### 2.7 Statistical analyses

We used *χ* ^2^ tests to analyze the occurrence of sea ducks in wetlands relative to expected proportions based on three metrics of availability (wetland surface area, and invertebrate biomass densities in cores and sweeps). More complex approaches that account for individual or spatiotemporal variation were not suitable, given our broad assessment. For example, we did not sample use of wetlands by individual females, or use of individual wetlands relative to their respective invertebrate biomass and instead used mean biomasses for each wetland type. We used the proportionate availability of wetlands and invertebrate biomass (relative invertebrate biomass density in each wetland type relative to the total across all wetlands) to calculate expected values of occurrence in these habitats by sea ducks. Values that differed statistically from the expected value indicated selection for or avoidance of that wetland type, relative to its availability. Values for these analyses were pooled across years, as we had inconsistent sampling effort (e.g., number of observers, Table A1) across all years of this 21‐year study, and our objectives focused on broad‐scale patterns of occurrence more than interannual variation in these patterns. Wetland frequency and surface area may have changed during our study period, yet we lacked annual data to assess such variation. Further, our estimates of prey density in 2017–2018 are assumed to indicate broad patterns of biomass among wetland types, despite probable variation among years. We tested associations between invertebrate biomass density and wetland types with Permutational Analysis of Multivariate Dispersions (PERMDIST) and Permutational Analysis of Variance (PERMANOVA), following recommendations of Clarke and Gorley (2015). We created resemblance matrices with Bray–Curtis dissimilarity, from which all PERMANOVA comparisons of invertebrate density were completed using wetland type and unique wetland number as model factors. PERMDIST and PERMANOVA comparisons were completed in Primer 7 (Quest Research Ltd) with log(*x* + 1) transformations to minimize issues with zero‐inflated data.

Fractions of time devoted to different behaviors are inherently non‐independent, as total proportions of each behavior type must sum to one. Therefore, parametric statistical approaches such as ANOVA or linear models were not appropriate. We used non‐parametric approaches (Spearman's rank‐order correlations, Kruskal–Wallis *H* test, and PERMANOVA) for all analyses of behavioral and prey data, as these approaches are more robust with such data (Hollander et al., 2015). Spearman's correlations and Kruskal–Wallis tests were completed in program R 4.0.2 (R Core Development Team, 2021). We tested for correlations among the four major behavior groups (foraging, loafing, locomotion, and reproductive behaviors), and among those behaviors and day of year, with Spearman's *r* ^2^. As our objectives for this study were to examine strong and well‐supported relationships, and behavioral data are inherently variable, we considered only correlations with *r* ^2^ ≥ .2 and *p* < .05.

### 2.8 Permitting and animal care

All data collection related to animals was conducted under applicable municipal, state, and federal permits, including a U.S. Fish and Wildlife Service permit for the capture and handling of federally threatened species. All methods were approved by the USFWS Region 7 Institutional Animal Care and Use Committee.

## 3 RESULTS

### 3.1 Relative area and duck use of wetland types

Surface area per wetland, and proportions of total wetland area, varied widely among wetland types (Table 2). Shallow *Arctophila* ponds were quite small and comprised the lowest percentage of total wetland area (1.3%). Deep *Arctophila* ponds were substantially larger than both Shallow *Arctophil*a and Shallow *Carex*, but the much greater abundance of very small Shallow *Carex* ponds resulted in a comparable proportion of total area (5.2%) to Deep *Arctophila* (4.8%), with both Shallow *Carex* and Deep *Arctophila* ponds comprising a far greater fraction of wetland area than Shallow *Arctophila*. Deep Lakes (Deep *Carex* and Open Lakes combined), because of their large size, had by far the greatest percentage of total surface area (78.6%), whereas Streams occupied a comparatively low proportion (10.1%).

**FIGURE 2:**
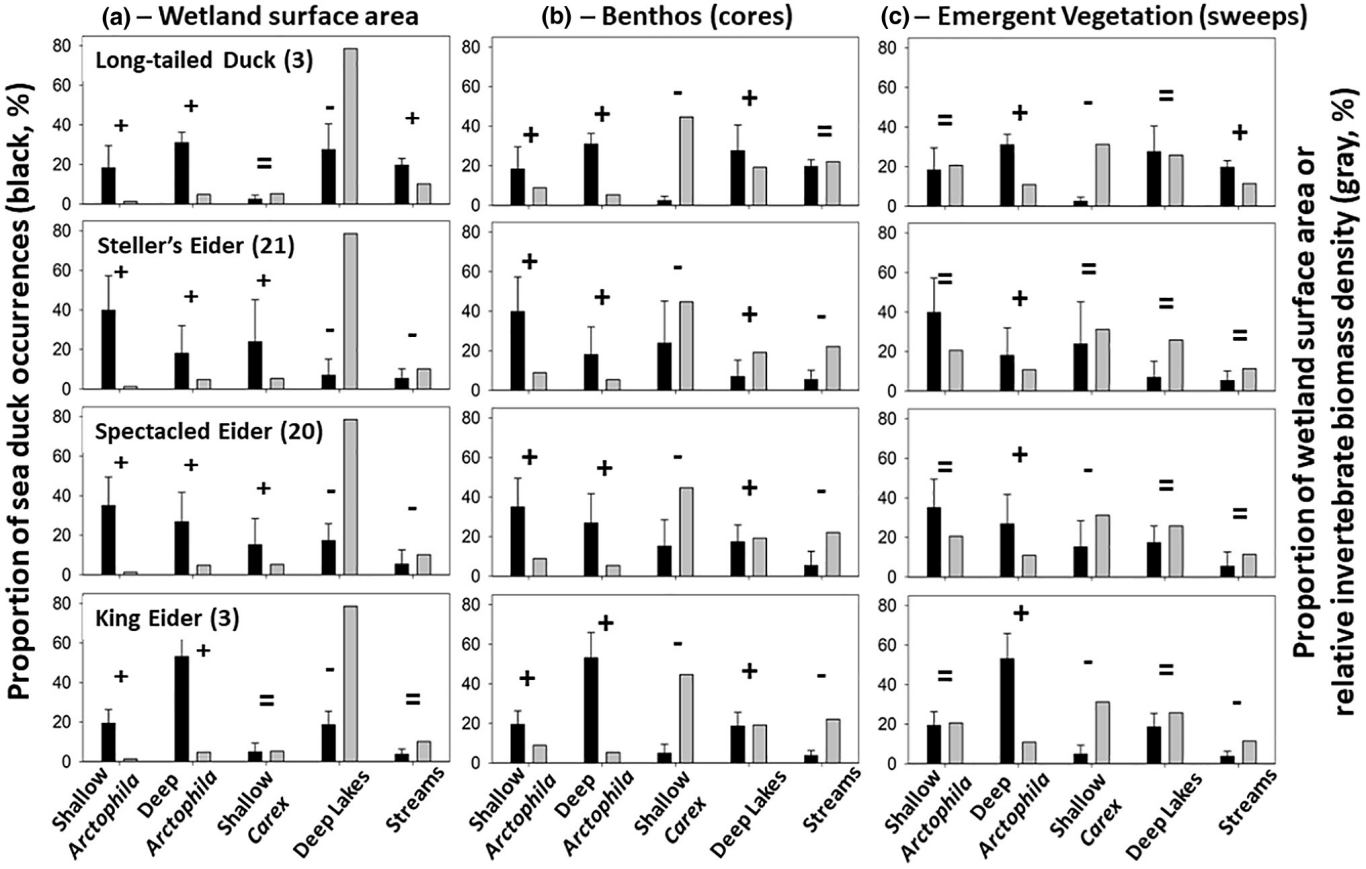
Percent occurrence of female sea ducks (black bars, interannual means in mid–late June 1999–2019 ± SD) versus (a) aggregate percent availability of wetland types in terms of total area (gray bars for different wetland types), or relative invertebrate biomass density in (b) cores (mg C/m^2^ of benthic surface area) and (c) sweeps (mg C/m^3^ of water column within emergent vegetation). Numerical values for wetland use relative to surface area are in Tables 2 and A5. Numbers in parentheses indicate the number of years for which habitat use was sampled for each species. Selection trends are indicated (+, use > availability; =, use equal to availability; and –, use < availability [*χ* ^2^, *α* = 0.05]).

In terms of surface area available, shallower wetlands were strongly preferred by all sea ducks relative to Deep Lakes (Figure 2a), except that long‐tailed ducks and king eiders used Shallow *Carex* equal to availability. Shallow and Deep *Arctophila* wetlands combined received far more use than Shallow *Carex* wetlands, despite much greater numbers of Shallow *Carex* ponds on the landscape (Table 2). Streams were also favored relative to Deep Lakes, but generally received far less use by eiders than did the other wetland types (Figure 2a). Long‐tailed ducks differed from eiders in greater use of Streams but lower use of Shallow *Carex*. Collectively, these results indicate stronger preference for and greater overall use of *Arctophila*‐dominated wetlands relative to their availability on the landscape, compared to Shallow *Carex*, Deep Lakes, or Streams.

### 3.2 Invertebrate biomass density and duck use of different wetland types

Total biomass density of invertebrates (excluding Oligochaeta, see Section 2.5) did not differ among years (PERMANOVA, *p* = .20 for benthic cores and .18 for net sweeps), and was not correlated with sampling date (June–July) for cores (*r* ^2^ = .04, *p* = .18) or sweeps (*r* ^2^ = .02, *p* = .33). Relative biomass density of invertebrates was greatest in Shallow *Carex* wetlands for both cores and sweeps (Figure 2b,c, Table A6). Selection, avoidance, or lack of preference for different wetland types relative to total invertebrate biomass in those types was quite consistent among sea duck species. In particular, despite the highest invertebrate biomass density in Shallow *Carex* ponds, proportionate use of Shallow *Carex* by all duck species was far less than the proportion of total invertebrate biomass that occurred in that wetland type. For core samples, use of both Shallow and Deep *Arctophila* by all duck species far exceeded that expected based on invertebrate biomass density. For invertebrate biomass density in emergent vegetation (net sweeps), use of Shallow *Arctophila* was roughly proportionate to density, whereas use of Deep *Arctophila* was far greater than relative density. For biomass density in emergent vegetation, duck use of Shallow *Arctophila* and Deep Lakes was the same as expected, use of Deep *Arctophila* was greater than expected, and use of Shallow *Carex* was mostly less than expected. Use of Streams was more variable among species, with long‐tailed ducks using streams greater than expected, king eiders using them less than expected, and Steller's and spectacled eiders using them as expected relative to invertebrate biomass density in emergent vegetation.

Chironomidae larvae comprised the largest and most consistent fraction of available relative biomass density (67%–85% in cores, and 50%–74% in sweeps; Table 3), with no other single taxon comprising ≥20% of total biomass density. This pattern of relative total biomass differed from the pattern in diets of adult female sea ducks (Miller et al., 2022). Insect larvae (mostly chironomids) comprised only 4%–14% of protein sources for egg production but comprised 48%–90% of invertebrate biomass in cores and 47%–82% in sweeps (Table 3). Crustaceans (mostly copepods) comprised a greater fraction of protein sources in diets (6%–34%) than did insect larvae, but still a lower fraction than was available (3%–19% in cores and 3%–13% in sweeps). Carbon content per individual insect larva (~80.5%–3196.5 μg C) was substantially higher than for the much smaller crustaceans (~20.6 μg C; S5 table in Plesh et al., 2023). This difference suggests that feeding on insect larvae was more profitable.

**TABLE 3:** Percent composition (mean ± SE) of relative C density of invertebrate prey taxa in different wetland types.

Taxon	Shallow *Arctophila*	Deep *Arctophila*	Shallow *Carex*	Deep Lakes	Streams
Benthic cores					
No. of wetlands	14	10	11	11	6
Crustacea	10.8 ± 16.9	14.8 ± 17.3	18.8 ± 21.2	4 ± 2.9	2.8 ± 6.9
Physidae snails	4.2 ± 10.6	5.2 ± 13.4	6.1 ± 13.3	0	7.4 ± 16.9
Insect larvae					
Chironomidae	70.2 ± 19.6	74.5 ± 31.9	66.7 ± 22.3	43.2 ± 3.4	73 ± 38.3
Other Diptera	0 ± 1.2	5.2 ± 16.6	0	0	0
Coleoptera	8.3 ± 13.6	0	2.1 ± 4	0.2 ± 0.2	15.3 ± 37.4
Plecoptera	0.3 ± 1.2	0.2 ± 0.8	0.9 ± 2.8	1.8 ± 1.0	1.5 ± 3.7
Trichoptera	6.3 ± 13.3	0	5.4 ± 10.7	2.8 ± 2.3	0
Net sweeps					
No. of wetlands	12	10	11	11	6
Crustacea	9.2 ± 10	13.2 ± 15.1	11.7 ± 15.8	5.0 ± 3	3 ± 4.1
Physidae snails	8.7 ± 14.1	12.1 ± 13.3	11.6 ± 15.9	1.5 ± 0.9	15.3 ± 21.3
Insect larvae					
Chironomidae	50 ± 22	57.3 ± 21.6	54.6 ± 26.7	29.3 ± 3.4	60 ± 27.2
Other Diptera	0.1 ± 0.2	1.9 ± 4.6	0.1 ± 0.3	0	0
Coleoptera	14.7 ± 11.6	2.4 ± 3.2	8.5 ± 16	5.1 ± 2.0	14.5 ± 13.2
Plecoptera	1.9 ± 3.1	2.5 ± 3.5	2.4 ± 7	6.6 ± 2.9	3.6 ± 5.3
Trichoptera	15.3 ± 10.9	10.5 ± 16.6	11.2 ± 16.6	5.8 ± 2.3	3.6 ± 4.2

*Note*: Values in each column for net sweeps and benthic cores separately may not sum to 100%, as values are means among individual wetlands and not global means. High SE for percentages averaged among individual wetlands, some of which contained none of a given taxon in samples, may exceed mean values. Deep Lakes are Deep Open Lakes and Deep *Carex* combined (see Section 2.4).

These results indicate that total biomass density of invertebrates, or the relative density of different taxa, may not be the predominant driver of wetland type selection. Other aspects such as slow thawing of sediments, or accessibility of invertebrates in macrophyte foliage or litter, potentially have important effects on availability of different prey. In particular, wetlands dominated by *Arctophila*, and especially Deep *Arctophila*, were used in greater proportion than expected based on total invertebrate biomass density and the proportion of insect larvae and crustaceans in different wetlands.

### 3.3 Behaviors of female sea ducks

We observed female behaviors of 624 long‐tailed ducks, 331 Steller's eiders, 88 spectacled eiders, and 153 king eiders, totaling 5980 min of observation time. We censored 8 records in which focal females were out of sight for ≥2 total min during an observation period. Fewer individuals were sampled in 2019 versus 2017–2018, due partly to heavy snowpack limiting road access and more rapid thaw. Most (33%–79%) behavior observations occurred in *Arctophila*‐dominated wetlands with low numbers in other wetland types, prohibiting comparisons of behaviors among wetland types. We did not detect appreciable variation in the proportion of time allocated to each behavioral category in 2017 versus 2018, the 2 years for which we had data on all four species (Kruskal–Wallis *H*, all *p* > .14), so we pooled data among years for all analyses. Across years, time spent in different behavioral categories did not shift appreciably with sampling date (all *r* ^2^ *<* .01, *p* > .05).

**FIGURE 3:**
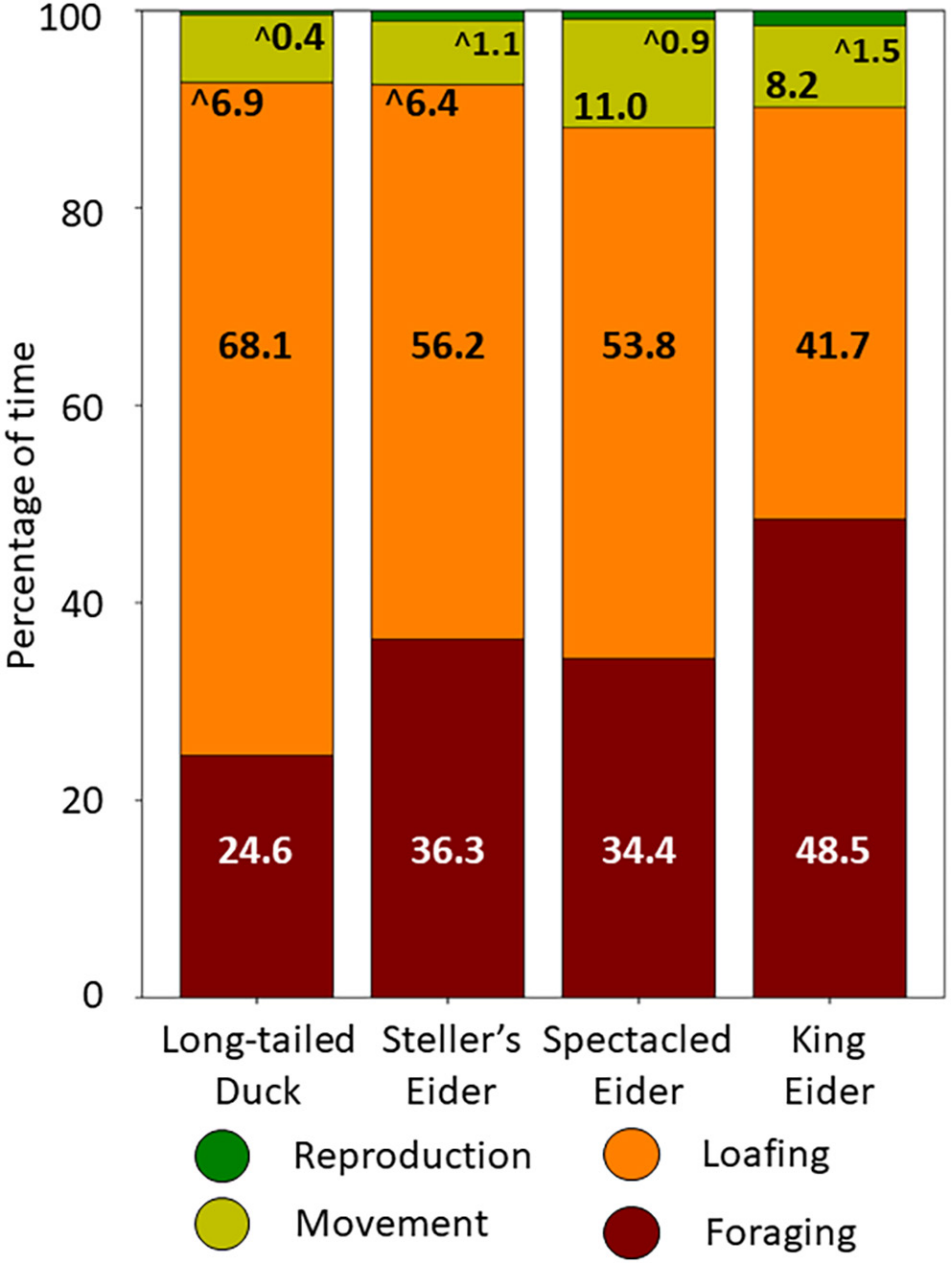
Percentages of time spent in different activities by female sea ducks during the prebreeding period, 2017–2019. Values are annotated for each broad behavioral category, rescaled after removing time spent out of view (see Section 2.6); values annotated with ^ indicate the category immediately above the annotated value (e.g., reproductive behaviors of long‐tailed ducks). Values may not sum to 100% due to rounding. Means and SD are shown in Table A7, and specific behaviors within major categories are in Figure A4.

**FIGURE A4:**
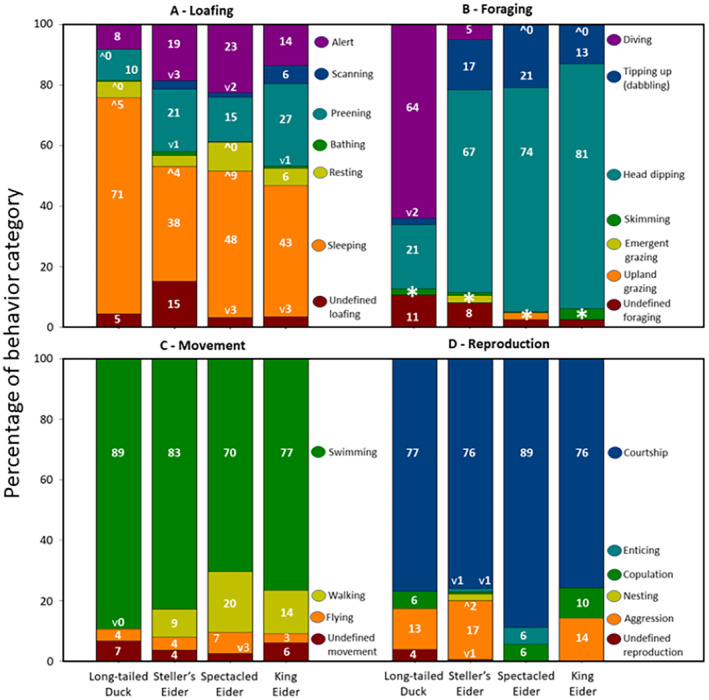
Category‐specific breakdown of specific behaviors by focal sea duck females. Annotated values are percentages of a specific behavior comprising the overall category (generalized activity budgets are in Figure 3). Asterisks indicate very rare behavior categories (≤1%) not otherwise annotated. Means and standard deviations are in Table A7. Values may not sum to 100% due to rounding.

Between arrival on the breeding grounds and onset of nesting, female sea ducks foraged almost entirely (89%–98%) within wetlands as opposed to surrounding uplands. Female sea ducks allocated 25%–49% of their daily time to foraging and 42%–68% to loafing, with less for movement (6%–11%) and reproduction (<2%) (Figure 3). Within each behavior group, 1–2 more specific behaviors dominated across species (Table A7, Figure A4). Foraging was mainly by head‐dipping (67%–81%) versus diving (<5%) in eiders, but mainly by diving (64%) versus head‐dipping (21%) in long‐tailed ducks. Eiders foraged mainly on prey accessible from the water surface (tipping up, head dipping, and skimming). Very few dives by spectacled and king eiders were observed (Table A8). Loafing involved mostly sleeping (38%–71% of loafing behaviors), movement behaviors mostly swimming (70%–89%), and reproductive behaviors mostly courtship (76%–89%) (Figure A4).

In summary, almost all of the ducks were paired upon arrival, so that very little time was devoted to courtship or other reproductive behaviors. Sea ducks used tundra wetlands almost entirely for feeding, or for loafing between feeding periods.

## 4 DISCUSSION

Relative occurrence of sea ducks on different wetland types varied somewhat among species, but the overall pattern was preference for shallow wetlands over Deep Lakes, and preference for Shallow and Deep *Arctophila*‐dominated ponds over Shallow *Carex* ponds despite the latter type's much greater abundance and total surface area. Although all duck species spent most of their time either feeding or resting between foraging bouts, their occurrence on different wetland types did not correspond to the biomass density of important diet items or of all invertebrates combined. For sea ducks that mostly feed during the prelaying period, it appears that *Arctophila*‐dominated wetlands are especially important, perhaps because of greater accessibility (as opposed to biomass density per se) of invertebrate prey. As explained below, these *Arctophila* wetlands are exceptionally vulnerable to the effects of climate warming of tundra landscapes.

### 4.1 Relative use of wetland types

Female sea ducks used a diversity of wetland types, but most occurred in Shallow and Deep *Arctophila*‐dominated wetlands (49%–73%). Occurrences were far lower in Shallow *Carex*, Deep Lakes, and Streams. Preferential use of *Arctophila* ponds was striking given that *Carex*‐dominated wetlands had a far greater surface area on the landscape and had greater biomass density of invertebrate prey.

Our results are roughly similar to prior studies of sea duck habitat use, with some notable exceptions. Bergman et al. (1977), working near Prudhoe Bay, Alaska, observed 52%–90% of prebreeding long‐tailed ducks, spectacled eiders, and king eiders in *Arctophila* wetlands, versus 49%–73% of females in our study. Bergman et al. (1977) did not distinguish use by males versus females; however, in our study, almost all sea ducks were paired upon arrival at the breeding site, so wetland use was likely similar for females and males. Bergman et al. (1977) also documented minimal use of Deep Open Lakes, compared to 17%–28% of duck occurrences in our study. Derksen et al. (1981) reported habitat use by waterbirds across the Arctic Coastal Plain; while the availability of each wetland type varied spatially, patterns followed those of Bergman et al. (1977) and our study with long‐tailed ducks and king eiders selecting Deep *Arctophila* wetlands.

In other studies, wetlands were either not classified consistently or included wetland types lacking in our study area. For example, Bart and Earnst (2005) documented habitat use of spectacled eiders on the Colville River Delta, where most wetlands were former river channels and thus were not comparable to those in our study. However, 33% of indicated spectacled eiders were in polygon ponds or complexes, and another 38% were in drained lake basins (“basin marsh” of Bergman et al., 1977). For spectacled eiders, 77% of occurrences in our study were in Shallow and Deep *Arctophila* and Shallow *Carex*. We did not distinguish the use of basin marsh habitats from the discrete wetlands that remain (e.g., Deep *Arctophila*) when basin marshes drain seasonally, but the general trend of 77% of spectacled eider occurrences versus 71% on the Colville Delta suggests that habitat use by spectacled eiders may be similar despite regional variation in wetland types and availability. Furthermore, despite major changes in the availability and surface area of wetlands from the 1940s to 2010s (Andresen & Lougheed, 2015), female sea ducks appeared to use the same wetland types in similar proportions as in other regions of the Arctic Coastal Plain.

It is possible that our strategy of delineating wetlands with remote sensing data only from 2012 to 2018 might have influenced our estimates of wetland coverage, but the gap in sampling likely has minimal impact on the relative patterns among wetland types. We used 2018 imagery (IfSAR) to delineate smaller wetlands, which are becoming smaller and less frequent on the landscape (Andresen & Lougheed, 2015). Given that all field sampling occurred ±1 year from the date the imagery was collected, our estimates of surface area and wetland perimeter for smaller wetlands are likely quite accurate. We did not delineate Deep lakes with ifSAR data due to persistent ice cover into late June when this imagery was collected. To avoid biases associated with ice cover, we instead delineated large lakes and streams with the vegetation model derived from 2012 WorldView satellite imagery (Andresen et al., 2017; Lara et al., 2016). Surface areas of these waterbodies were orders of magnitude larger than ponds. While estimated surface area and wetland perimeter may differ slightly from actual values due to changes over 6 years, proportional changes over time in these much larger wetlands are likely minimal relative to changes in smaller wetlands. Thus, our use of combined datasets may somewhat overestimate the area of Deep Lakes but should have minimal impact on the relative sizes of different wetland types. Indeed, even if our estimates of large lakes were biased by nearly twice the actual value, the overall patterns relative to occurrence would not differ. For Streams, we also used the vegetation model to manually delineate seasonally inundated vegetation adjacent to streams, as streams fluctuate seasonally in surface area far more than the other wetland types.

While our occurrence data for Steller's and spectacled eiders date back to 1999, our data on king eiders and long‐tailed ducks were collected from 2017 to 2019. Using the most recent remotely‐sensed data for the smaller wetlands could potentially result in some biases for the earliest samples, yet we lack data to infer any potential changes in wetland availability from the earliest years of this study to the last. We can, however, make some predictions using the linear model of wetland surface area developed by Andresen and Lougheed (2015), which suggests that wetland surface area and number did not change statistically from 2002 to 2013. Thus, while there is potentially some bias given the range of years in our wetland occurrence data versus the remotely‐sensed data, such bias is within the range expected of interannual variation independently of directional change.

### 4.2 Variations in prey density

Carbon biomass varied widely among wetlands of each type, and particularly among types. Carbon is a widely‐used metric of energy in food webs, and we used it here instead of energy content of specific individual prey. Such wide variability, especially among wetlands of the same type, may mask patterns in use relative to prey biomass that might be relevant to individual birds. We did not account for individual variation among female sea ducks, instead using aggregate numbers in each wetland type. Telemetry studies that account for individual variation among birds may yield different results than we see at a coarser scale and indeed might reveal preference by certain females for certain wetlands that provide greater prey biomass. However, profitability of foraging is an important consideration, and may dictate appreciable deviation from expected patterns of occurrence based on prey biomass alone.

Prey is not expected to be universally distributed within or among wetlands. Patches of prey, such as in stands of emergent *Arctophila*, may be highly profitable and meet energetic needs of female sea ducks during the prebreeding period. However, sea ducks lack omniscience as to where profitable prey are, and must therefore expend energy and time seeking it out. Indeed, during behavioral observations, foraging females either stayed in the same general location while feeding or worked along wetland edges or through emergent vegetation for the duration of each foraging bout. This pattern might be interpreted as either capitalizing on known resources (not moving) or searching for new profitable patches. Other factors, such as the proximity to nesting habitats, may also influence site selection, and females using specific wetland types may be choosing sites adjacent to wetlands that provide known patches of prey. Hens take breaks during incubation, and adjacent wetlands likely provide resources. How the availability and profitability of prey dictates individual decisions warrants further examination via telemetry studies at much finer scales than we could consider in this study.

### 4.3 Accessibility of prey

Our estimates of invertebrate biomass density varied widely among individual wetlands, with the greatest mean biomass density in Shallow *Carex* wetlands. Invertebrate community structure within wetland types may not change with climate warming (Lougheed et al., 2011). However, increased loading of nutrients and dissolved organic matter from thawing tundra may be increasing microalgal and bacterial production and thereby increasing invertebrate production (Plesh et al., 2023; Reyes & Lougheed, 2015). Such increases may vary in effects on sea ducks depending on methods by which the different duck species access those prey.

For example, in tundra ponds, eiders forage nearly exclusively from the water's surface, whereas long‐tailed ducks forage mainly by diving (Figure A4). Thus, in deeper wetlands, only prey accessible from the surface (~30 cm depth) are available to eiders via the foraging modes we observed, whereas all prey within the wetland are available to long‐tailed ducks. All the duck species we studied are adept divers, so why eiders avoid diving in freshwater wetlands is unclear, especially when eiders forage by diving in freshwater ponds elsewhere (e.g., Solovieva, 2005; N. R. Graff, personal observation). One potential explanation is that all the resources they require are accessible from the surface in vegetated wetlands. Such access would be most pronounced in wetlands dominated by *Arctophila*, which grows as both an emergent and submersed plant with a near‐surface canopy throughout many wetlands, as opposed to *Carex* which grows only as an emergent, often only near shorelines. The diets of long‐tailed ducks included high fractions of Oligochaetes and sticklebacks (Miller et al., 2022), which both may be more accessible in the benthos and water column. Eiders, especially Steller's eiders, also had substantial dietary inputs from emergent plants (6%–32% of proteins), which may be ingested incidentally while accessing invertebrate prey on vegetation.

Variations in foraging habitat use by eiders have been observed in other areas of Arctic tundra. For example, in the Lena Delta along the Russian Siberian coast, Steller's eiders devoted over 30% of foraging time to feeding in uplands (“foraging from moss” in Solovieva, 2005), which we very rarely observed. During the prebreeding period near Teshekpuk Lake, roughly 100 km southeast of Utqiaġvik, king eider females foraged ~30% of each day by surface feeding (Oppel et al., 2011), compared to almost 50% in our study. These variations indicate a measure of flexibility depending on food availability.

### 4.4 Importance of *Arctophila* wetlands

During June and early July, emergent *Arctophila* and *Carex* were senesced and at annual minima. *Arctophila*‐dominated wetlands were used by female sea ducks far more than expected, as *Arctophila* wetlands comprised a very low proportion of total wetland surface area. Prey biomass in *Arctophila* wetlands was moderate for both cores and sweeps – greater than that in Deep Open Lakes and Deep *Carex*, but less than in Shallow *Carex* wetlands. However, *Arctophila* wetlands accounted for 49%–73% of occurrences of female sea ducks, versus 24%–32% in *Carex*. Why, then, are *Arctophila* wetlands used so much more than *Carex*?

In our study area (and throughout the Arctic Coastal Plain; Bergman et al., 1977; Derksen et al., 1981), wetlands are consistently fringed with *Carex* (except for Deep Open Lakes). Even in Deep *Carex* wetlands, *Carex* grows only in shallow portions of the wetland and on the periphery, while the center of the wetland is unvegetated. During our study period (during and shortly after snowmelt), senesced *Carex* was in dense peripheral stands and mostly submerged by flooding, but lodged material was still present in the water. *Arctophila* wetlands also have peripheral stands of *Carex* but with emergent and even submersed *Arctophila* between that fringe and the open portion of the wetland (e.g., figure 7 in Andresen & Lougheed, 2015). *Arctophila* grows in much deeper water (20–80 cm) than does *Carex* (<30 cm; Bergman et al., 1977), but in less dense stands that were also senesced and lodged during our study. Senesced *Arctophila* covers only a small portion of some wetlands but almost completely covers others (Figure 1c).

During our study period, most surface‐based foraging by ducks occurred within senesced stands of submersed vegetation (mostly *Arctophila*), where the benthos could not be accessed without diving. Pond bottoms beneath *Arctophila* stands include a layer of unconsolidated detritus from prior years, whereas sediments beneath *Carex* contain a tight mass of roots; substrates beneath *Arctophila* may facilitate easier foraging relative to the dense root masses of *Carex*. *Arctophila* stems and leaves also float after senescence, forming a near‐surface substrate for algae, bacteria, and the invertebrates that consume them. We observed many females with vegetation (presumably *Arctophila*) in their bills while foraging, although we saw no evidence of grazing upon vegetation directly.

In this study, we did not document the biomass of meiofaunal oligochaetes. The range of sizes of oligochaetes was not retained consistently by our 0.5‐mm sieve, which is standard for sampling macroinvertebrates which typically comprise the prey of ducks. Oligochaetes were also broken down in our samples which were frozen for later isotopic analyses. We anecdotally observed oligochaetes in most sweeps of emergent vegetation, especially within emergent and detrital stands of *Arctophila*, yet we recovered very few oligochaetes in thawed samples. Subsequent isotopic analyses unexpectedly indicated that meiofaunal oligochaetes were important foods for female sea ducks in our study area, especially for egg production, for which they comprise 14%–52% of protein sources (Miller et al., 2022). A previous study found that oligochaetes comprised only ~7% of invertebrate abundance in tundra ponds of our study area (Butler et al., 1980). This low relative abundance and resulting low biomass of these small organisms would not have changed our results regarding total invertebrate biomass density, which may be a crude metric given apparent selection of certain prey taxa. Further work is needed to explore effects of foraging profitability for different potential prey on wetland‐type selection by sea ducks.

While this study focused only on use of wetlands during the prenesting period, *Arctophila* wetlands continue to be used by sea ducks throughout the summer. Indeed, within our study area in 2011–2012, 48%–50% of locations of Steller's eider broods, and 34%–47% of spectacled eider broods, occurred within *Arctophila* wetlands (Safine, 2013). For Steller's eiders specifically, in 6 years from 1995 to 2012, 43%–85% of brood locations were in wetlands dominated by *Arctophila* (Safine, 2013). Thus, *Arctophila* wetlands provide critical resources not only for females upon arrival at the breeding grounds but also for the rearing of young.

### 4.5 Long‐term shifts in wetlands and prey availability

In our study area, a sample of Deep *Arctophila* ponds initially sampled in the 1970s was resampled four decades later to assess long‐term changes in specific wetlands (Lougheed et al., 2011; McEwen & Butler, 2018). Benthic sediments of wetlands were not thawing earlier in the spring prebreeding period but were freezing later in the fall, while ice‐free periods and numbers of degree days had increased (McEwen & Butler, 2018). Water temperature, dissolved nutrients, and the biomass and spatial extent of wetland macrophytes also increased during this period (Lougheed et al., 2011). However, shifts in invertebrate community structure were minimal. Increased temperature, duration of the ice‐free period, and leaching of inorganic nutrients and dissolved organic matter from thawing tundra should all increase invertebrate production, so production of sea duck foods in particular wetlands should not decrease with climate warming (Plesh et al., 2023). However, increased evapotranspiration, expansion of shoreline emergent vegetation, and drainage via thawed channels in permafrost (taliks) appear to be decreasing the availability of small, shallow wetlands on the landscape. Indeed, in our study area, shallow wetlands <1 ha declined by 30% in area and 17% in number over 65 years from 1948 to 2013 (but mostly from 1948 to 2002; Andresen & Lougheed, 2015), and at a faster rate than larger or deeper wetlands. Similar trends have been reported throughout Arctic regions (Marsh et al., 2009; Smith et al., 2005; Smol & Douglas, 2007). These trends apply to both Shallow *Arctophila* and Shallow *Carex* wetlands, which account for roughly half of all sea duck occurrences in this study, with less rapid change in similarly important Deep *Arctophila* wetlands. Long‐term declines in breeding populations of sea ducks across the Arctic coastal plain of Alaska may be related to these changes.

It is important to note that the impacts of climate change on tundra wetlands, and species reliant on them, are expected to be highly variable both spatially and temporally. While in our study area, wetland number and area have declined (Andresen & Lougheed, 2015), novel wetlands are appearing elsewhere on the Arctic Coastal Plain as permafrost melts and water pools on the surface (Raynolds & Walker, 2016). The onset of spring progressed by ~16 days from 1992 to 2016 (Amundson et al., 2019). Spring conditions that no doubt influence the use of wetlands are also more variable now than in the past; for example, 2016 had one of the earliest springs, and 2018 the latest, among the 21 years of this study), so birds must adapt to both long‐term changes and very different conditions each year between arrival and onset of laying. Foraging on macroinvertebrate prey in tundra wetlands may offset the metabolic costs of migration and subsequent carry‐over effects on nutrition (Alisauskas & Devink, 2015) that impact population trajectories. Across the Arctic Coastal Plain, population trends of sea ducks are generally stable, albeit at greatly reduced levels, but vary both spatially and temporally (Amundson et al., 2019). Sea ducks are likely adapting to shifting conditions, but exactly how well they can adjust as climate change continues to modify their environments is unclear.

In conclusion, sea duck species in our study area preferred shallow wetlands dominated by *Arctophila*, despite higher density of invertebrate biomass in *Carex*‐dominated wetlands. This preference may result from greater accessibility of invertebrates in emergent, submersed, and detrital foliage of *Arctophila*, and perhaps the less root‐bound sediments beneath *Arctophila*. The preferred and highly used *Arctophila* wetlands appear to be especially vulnerable to loss via climate warming, especially Shallow *Arctophila*. Given the rapid and massive changes occurring in Arctic tundra environments, the impacts of such changes on sea ducks will depend on their flexibility in acquiring prey efficiently in other wetland types.

## AUTHOR CONTRIBUTIONS

**Micah W. C. Miller:** Conceptualization (lead); data curation (lead); formal analysis (lead); funding acquisition (supporting); investigation (lead); methodology (lead); writing – original draft (lead); writing – review and editing (lead). **James R. Lovvorn:** Conceptualization (equal); formal analysis (equal); funding acquisition (lead); investigation (equal); methodology (equal); project administration (lead); writing – original draft (equal); writing – review and editing (equal). **Nathan R. Graff:** Investigation (supporting); project administration (supporting); writing – original draft (supporting); writing – review and editing (supporting). **Neesha C. Stellrecht:** Funding acquisition (supporting); project administration (supporting); supervision (equal); writing – original draft (supporting); writing – review and editing (supporting). **Steven P. Plesh:** Investigation (supporting); writing – original draft (supporting); writing – review and editing (supporting).

## FUNDING INFORMATION

Funding was provided by the Fairbanks Field Office, Endangered Species Program of the U.S. Fish and Wildlife Service (Cooperative Agreement Award F20AC00328 to JRL), and the Arctic Science, Engineering, and Education for Sustainability program of the National Science Foundation, Office of Polar Programs (grant ARC‐1263051 to JRL).

## CONFLICT OF INTEREST STATEMENT

None declared.

## Data Availability

Data used in this study are available via the Dryad digital data depository: DOI: 10.5061/dryad.t1g1jwt7p.

## APPENDIX 1 DESCRIPTION OF GROUND‐BASED PAIR SURVEYS TO QUANTIFY OCCURRENCE OF STELLER'S AND SPECTACLED EIDERS (ADAPTED FROM GRAFF, 2021)

### 1.1

From 1999 to 2019, ground‐based surveys were completed within our study area to determine how many Steller's and spectacled eiders were present on the landscape, their sex distribution, and in what habitats were they located. Both species are listed as Threatened under the U.S. Endangered Species Act and were the focus of all aspects of the surveys.

#### Survey design and timing

The ground‐based foot survey was designed to be completed annually by a group of ~10 observers working in smaller groups (three to five people) and covering multiple segments of the larger ~175 km^2^ study area per day. Up to 30 discrete subareas (6.67 ± 2.1 km^2^ in area, Figure A1) were surveyed annually; a few have been removed from more recent surveys due to logistical constraints of accessing more remote areas of the study area. For example, the 2017 survey design is shown below (Figure A1). One to three subareas were surveyed daily in June, although in 2017 and 2018 late snowfall delayed the onset of the survey and surveys were completed in early July. The westernmost areas of the study area were completed first, as these tend to have greater vertical relief and snowmelt typically occurs earlier there than in the eastern portions of the study area. For many years, the eastern portion of the study area was inaccessible until the snow melted from roads that were not maintained during winter.

For these reasons, and because sea ducks were typically concentrated in a few large wetland basins until snowmelt was complete, surveys began once we observed that most snow had melted each spring. Surveys were not conducted when weather substantially limited visibility. Survey duration was typically 9–14 days. The total area surveyed did vary among years but typically was 172–192 km^2^. Survey duration, timing, and coverage are summarized in Table A1.

Within each subarea, observers walked at a standard distance apart throughout one side of the subarea before returning on the opposite side of each subarea. The distance between observers and paths taken varied depending on subarea size, the number of observers, or wetlands or other barriers along their path. While later surveys have recorded the actual paths walked by observers, such data are absent for most years. However, the objective in all years is to get 100% spatial coverage across the entire study area.

#### Data collection

For each observation of Steller's and spectacled eiders (and king eiders in 2017–2019 only), the following data were recorded:

1. *Location* – Earlier years of the study (1999–2010) used printed maps and paper field notes, with locations approximated by observers on maps and by handheld low‐precision GPS units (e.g., Garmin E‐Trex or similar units). In later years of the study (2011–2019) we used finer‐precision GPS (Trimble Juno or similar), with all data collection completed via forms in the device. In many cases, offset estimation was used to estimate where the bird was located relative to the observer, and locations were estimated using a range and bearing from the observer. We acknowledge the high spatial error associated with occurrence data in this study and have elected instead to use nonspatial approaches to remove this error from our dataset.
2. *Species –* The species was recorded to the best of the observer's ability. In some cases, it was not possible due to lighting or other factors to identify the individual to species, so a broader category was used, such as unknown eider (typically when it was not possible to distinguish female spectacled and king eiders).
3. *Count ‐* Most observations were of single birds or pairs, but at times, larger flocks were observed.
4. *Occupancy code* – For each observation, a categorical occupancy code was recorded as a metric of how likely that individual was associated with a nest near that location. In some cases, the code was updated if a bird shifted among categories; for example, a bird which initially passed over (5, e below), and then landed and began foraging in a wetland (4, d below) would be categorized as (4).
  - Confirmed nest – nest bowl with eggs, or an incubating female.
  - Probable nest – a nest is suspected based on female behavior, but not actually located. For example, a female flushed from the tundra or pond edge and remained close by, but no nest was observed.
  - Possible nest – behavior suggests a nest may be nearby, but without any precise indication of where. For example, a pair of eiders swims to the edge of a pond and remains there despite observers passing close by.
  - Present – Swimming or loafing in a wetland or shoreline, but without any further indication of nesting behavior.
  - Passing through – Bird which flew over the observers without landing.
5. *Habitat type –* For locations of eiders, a wetland habitat type was also recorded. We generally followed wetland habitat types of Bergman et al. (1977), modified to include other categories relevant to anthropogenic modifications of the landscape such as ditches and manipulated basins. Any individuals not clearly associated with an adjacent wetland, including females on nests, were considered to be in dry tundra. Habitat types are described in detail in Table A3.

For analyses in this paper, only the count, occupancy category, and wetland habitat type were used to create the occurrence dataset for each species. All procedures for data filtering and integration of various datasets (ground survey, behavioral observations, and incidental data) into a single dataset are described in Appendix 2.

## APPENDIX 2 QUALITY CONTROL AND DATA FILTERING FOR SEA DUCK OCCURRENCE DATA

### 2.1

We used multiple datasets of habitat use by our four study species, each with slightly different sampling methods. Most occurrence and wetland use data were from the U.S. Fish and Wildlife Service (USFWS) ground‐based survey in June 1999–2019 (Graff, 2021; described in detail in Appendix 1). Steller's and spectacled eiders were recorded in all years, and king eiders in 2017–2019. We also analyzed wetland use data from individual females (including long‐tailed ducks) monitored during behavior observations. Finally, some additional data on all species were collected incidentally by observers outside of focused surveys in 2017–2019.

Not all data were suitable for use, however, so we filtered our dataset to include only those data which fit the following criteria: (1) ≥1 female sea duck was observed; (2) females were within wetland habitats (not flying, in upland habitats, or on nests, based on occupancy codes in Appendix 1); and (3) wetland habitat was adequately described. While some earlier data lacked spatial locations, we did include any values for which the number of females and wetland type were recorded. Unfiltered and filtered data are summarized in Table A2.

## APPENDIX 3 DATA PROCESSING FOR WETLAND DELINEATION WITH REMOTE‐SENSING DATASETS

### 3.1

It was not feasible to measure characteristics of all wetlands used by sea ducks in our study. Instead, we relied on remote sensing to delineate wetland perimeters. We lacked a remote sensing dataset which classified wetlands into the same types we used for habitats of sea ducks, so we used ground‐based wetland classification to assign wetland types, and estimated surface area from remote sensing data.

Ground‐based wetland surveys were completed in 400 × 400 m plots (*n* = 24) randomly located within our study area. For logistical reasons, we selected plots within 2 km of roads or beaches (Figure 1b of main text). Within the spatial boundary of each wetland within each plot, a point was recorded via a handheld GPS (Trimble, Inc.) and the wetland classification recorded. During June–July 2017–2019, we classified 2668 tundra wetlands into six wetland types that generally follow the classifications of Bergman et al. (1977, wetland types described in Section 2.4 of main text). Field data were processed with Pathfinder Office (Trimble, Inc.) with ~5 m horizontal precision.

We used two remote sensing datasets to delineate the perimeters of individual wetlands across the entire Barrow Peninsula in the Arctic Coastal Plain (left panel of figure 5 in Andresen et al., 2017). The first, a vegetation community model (Andresen et al., 2017; Lara et al., 2016), was derived from WorldView satellite imagery (0.59‐m pixel resolution) taken in August 2012. This model classifies vegetation into discrete communities that follow a wetness gradient from open water to dry upland vegetation, from which pixel values 1 (open water) and 2 (aquatic graminoid vegetation) were considered as potential wetland polygons. Second, we used orthorectified inferometric serial aperture radar imagery (IfSAR or InSAR; U.S. Geologic Survey, 2021) at 0.625‐m pixel resolution to delineate smaller wetlands that were not adequately represented by the vegetation model alone. IfSAR imagery was collected in July 2018 and was more accurate for smaller wetlands than the vegetation model, but ice cover in larger lakes prohibited use for larger lakes and ponds. Remotely‐sensed datasets were processed in ArcGIS Pro 3.0 (ESRI).

Our processing workflow (shown graphically in Figure A2) to produce wetland surface areas from these rasters was as follows:

1. As the pixel resolution of the rasters differed, we first resampled the vegetation raster (0.59 m pixels) to the resolution of the IfSAR imagery (0.625 m pixels) using a nearest‐neighbor algorithm. Nearest neighbor algorithms provide superior resampling capacity with classified data relative to other resampling algorithms.
2. We reclassified pixel values into a binary format (wetland or non‐wetland) for the vegetation model and IfSAR data separately. For the vegetation model, raster values 1 (open water) and 2 (aquatic graminoid vegetation) were selected as wetlands, and all other values as non‐wetland. For the IfSAR dataset, we considered wetlands to be any pixels with values ≤30, based on values falling within the boundaries of *n* = 17 wetland perimeters mapped with handheld GPS units in 2017.
3. To create a single raster integrating both data sources, we applied a raster calculator to sum all wetland pixels into a raster for all wetland surface areas. Briefly, by converting to a wetland versus non‐wetland binary model, any values of 1 (wetland) will sum to a value greater than 0 (non‐wetland); we converted this summed raster into a final binary model with wetland (1) versus non‐wetland (0) values.
4. Using this final raster, we created polygons for individual wetlands by converting the raster into a feature layer. We then extracted the polygons within or intersecting the spatial boundary of our study area.
5. We calculated wetland surface area and then joined classified wetland locations to the corresponding wetland perimeter. However, not all wetland locations were adequately joined, and in some cases, data from multiple classified wetlands were joined to a single polygon. To minimize process error associated with linking classified wetland locations to polygons derived from remote sensing data, we assigned a threshold limitation of 7.5 m between GPS locations and nearby wetland polygons. Mean horizontal precision of GPS points was 5.01 m (95% CI 4.97–5.05), with an upper limit of the interquartile range of 7.85 m. The devices we used did not allow for post‐processing of locations to increase precision. Six locations with mean spatial precision >7.5 m were removed from our dataset, as were another 569 locations for which our remote sensing dataset failed to delineate any wetland polygons within 7.5 m, resulting in a final spatial dataset of 1085 classified polygons. To validate the accuracy of remotely sensed wetland perimeters, we compared surface area estimated by remote sensing and via handheld GPS. We found high agreement between the two metrics (paired *t*‐test, *p* = .237; Spearman's *R* ^2^ = .984, *p* = .033), showing that the remotely sensed values were representative of actual wetland areas.
6. For streams, we manually digitized the stream boundaries using the vegetation model described above. As streams may vary seasonally in the amount of water present, no previously described remote sensing dataset defines them effectively for our use. To delineate streams as consistently as possible with delineation of lakes and ponds, for streams we used the minimum flooded area, assuming that vegetation was a reliable indicator of seasonal flooding typical of streams. For all streams terminating in coastal areas, we removed the portion of streams that likely experienced marine brackish conditions, as such areas would have very different plant and invertebrate communities from other wetlands in this study; birds use of these brackish areas also tended to be very low (Micah W. C. Miller, personal observation). While our estimates for Streams are likely biased relative to the precision of the remotely‐sensed wetlands, the relative area of streams is likely representative of conditions in spring. Streams shifted in extent seasonally far more than any other wetland type, so we feel that manual delineation is sufficient for our objectives at a landscape scale.

**TABLE A1:** Summary of ground‐based survey effort for quantifying occurrence of sea ducks within wetlands, 1999–2019.

Year	Area (km^2^)	Subareas	Start	End	Duration (days)	Observers (*n*)
1999	172	30	14 June	25 June	12	3–7
2000	136	30	14 June	29 June	15	3–6
2001	178	30	15 June	27 June	12	3–5
2002	192	27	11 June	20 June	9	3–4
2003	192	30	12 June	20 June	8	3–5
2004	192	30	14 June	23 June	9	3–5
2005	192	30	16 June	26 June	10	2–5
2006	192	30	12 June	21 June	9	3–6
2007	136	21	12 June	18 June	6	2–5
2008	167	26	13 June	24 June	11	3–7
2009	170	27	12 June	20 June	8	3–4
2010	176	27	16 June	25 June	9	3–8
2011	180	28	12 June	21 June	9	2–4
2012	178	26	11 June	20 June	9	2–6
2013	178	29	10 June	20 June	10	2–6
2014	178	25	15 June	26 June	11	2–5
2015	172	29	12 June	22 June	10	3–6
2016	172	30	9 June	20 June	11	3–10^a^
2017	172	27	20 June	2 July	12	3–10^a^
2018	172	27	28 June	9 July	11	4–13^a^
2019	172	26	17 June	1 July	14	3–12^a^

*Note*: Surveys were initiated at comparable phenology relative to snow melt across years. Surveys were completed via daily coverage of one to three subareas 6.67 ± 2.1 km^2^ in area.

^a^ In 2016–2019, a single subarea was surveyed with a large group of observers, but observer number for all other subareas was consistent with other years of this study.

**TABLE A2:** Sea duck occurrence data summary, 1999–2019.

Species	Occurrence surveys (1999–2019)	Behavior observations (2017–2019)	Incidental (2017–2019)	Total occurrences (All years)
Unfiltered data				
Long‐tailed duck	Not recorded	624	803	1427
Steller's eider	862	331	329	1522
Spectacled eider	672	88	101	861
King eider	69 (2017–2019 only)	153	203	425
Filtered data				
Long‐tailed duck	Not recorded	621	803	1424
Steller's eider	814	329	182	1325
Spectacled eider	543	88	63	694
King eider	33 (2017–2019 only)	153	170	353

*Note*: Values represent individual observations of female sea ducks. The numbers of females observed in each wetland type were summed to estimate the total frequency of use (main text, Figure 2). The upper panel includes unfiltered data, and the lower panel includes the final filtered dataset.

**TABLE A3:** Wetland classifications used in this study (Bergman et al., 1977) compared to the classification of Cowardin et al. (1979).

Bergman et al. (1977)		Cowardin et al. (1979)				Wetland characteristics	
Wetland type	Class	System	Subsystem	Class	Subclass	Dominant vegetation	Description
Flooded Tundra	I	Palustrine	None	Emergent Wetland	Persistent	Any	Seasonally‐flooded tundra, following rain or snowmelt events
Shallow *Carex*	II	Palustrine	None	Emergent Wetland (Unconsolidated Bottom)	Persistent (sand/organic)	*Carex*	Depth <40 cm with few open sections of water
Shallow *Arctophila*	III	Palustrine	None	Emergent Wetland	Non‐persistent	*Arctophila*	Depth <40 cm with few open sections of water
Deep *Arctophila*	IV	Palustrine (lacustrine)	None (limnetic littoral)	Emergent Wetland (unconsolidated bottom)	Non‐persistent (sand/organic)	*Arctophila*	Depth ≥40 cm with ≥1 large section of open water
Deep Open Lake	V	Lacustrine	Limnetic	Unconsolidated bottom	Organic (sand)	None	Large lakes with little to no emergent vegetation
Basin Complex	VI	^a^	^a^	^a^	^a^	Any	Large wetland complexes flood seasonally but differentiate as waters recede. Typically, wetlands within basin complexes are of various types
Beaded Stream	VII	Riverine	Lower Perennial	Emergent wetland	Non‐persistent	Any	Flowing water with deep pools and runs
Coastal Marsh	VIII	Estuarine	Intertidal	Emergent wetland	Persistent	*Carex*	Small, shallow wetlands regularly inundated by tides or storm events, with low, salt‐tolerant vegetation, e.g., *Carex subspathea*
Deep *Carex*	^b^	Palustrine (lacustrine)	None (limnetic littoral)	Emergent Wetland (unconsolidated bottom)	Non‐persistent (sand/organic)	*Carex*	Depth ≥40 cm with ≥1 large section of open water
Ditch	^b^	Variable	Variable	Variable	Variable	Any	Non‐flowing, linear features, often associated with historical oil and gas development
Manipulated Basin	^b^	Variable	Variable	Variable	Variable	Any	Artificial wetlands created by humans (drainage ponds, impoundments)

*Note*: Adapted from Table 1 in Derksen et al. (1981). Terms in parentheses for the Cowardin classification are included to avoid ambiguity for some wetland types.

^a^ Basin complexes contain multiple smaller wetlands of various types, and are present only early in the season, after which water levels recede and smaller wetlands become distinct from one another. No equivalent wetland type exists in the Cowardin et al. (1979) classification system.

^b^ Deep *Carex* wetlands, ditches, and man‐made basins were not included in the Bergman et al. (1977) classification but were added, as these habitat types are present in our study area and may be used by sea ducks. Analyses apply only to the use of non‐anthropogenic wetland types. Pair survey data from 1999 to 2019 did not differentiate Deep *Carex* wetlands from Deep Open Lakes, so these are grouped as Deep Lakes in all analyses.

**TABLE A4:** Behavior classifications for prebreeding sea ducks used in focal individual sampling.

Category	Behavior	Description
Loafing	Alert	Alert, actively scanning for predators
	Scanning	Looking around, but not actively alert
	Preening	Preening, stretching wings or legs, or ruffling feathers
	Bathing	Bathing, except for bathing associated with copulation
	Resting	Resting, head low but not tucked under wing
	Sleeping	Sleeping, head tucked under wing
	Loafing	Other loafing behaviors
Foraging	Diving	Diving, entire body submerged
	Tipping up	Tipping up, head submerged and rear end out of water
	Head dipping	Head intermittently submerged but the body on the surface of water
	Skimming	Skimming water surface, bill in water but head and body on surface
	Emergent grazing	Grazing in emergent vegetation, usually by walking
	Upland grazing	Grazing in upland habitats away from water
	Foraging	Other foraging behaviors
Movement	Swimming	Swimming
	Walking	Walking in very shallow water or on tundra
	Flying	Flying, including prospecting flights
	Movement	Other movement behaviors
Reproduction	Courtship	Courtship behaviors such as head shakes, cooing, or calling
	Enticing	Female soliciting copulation
	Copulation	Copulation and post‐copulation bathing
	Nesting	Any nesting behavior (nest‐building, laying eggs, etc.)
	Aggression	Aggression toward interloping males or other females
	Reproduction	Other reproductive behaviors
Out of view	Out of view	Bird not visible

*Note*: The category “Out of view” was used when a focal hen was not visible for some portion of the 5‐min observation period.

**TABLE A5:** Patterns of occurrence (interannual mean % of total observations ± SD) of sea ducks on tundra wetlands, 1999–2019.

Wetland type	Long‐tailed duck	Steller's eider	Spectacled eider	King eider
No. of years	3	21	20	3
Shallow *Arctophila*	18.2 ± 11.3 A, a	39.8 ± 17.5 A, b	35 ± 14.4 A, ab	19.4 ± 6.9 A, a
Deep *Arctophila*	31 ± 5.3 A, ab	18 ± 14 BCD, b	26.8 ± 14.9 AB, ab	53.1 ± 12.8 B, a
Shallow *Carex*	2.4 ± 2.1 B, a	23.8 ± 21.3 AC, b	15.1 ± 13.3 B, ab	4.9 ± 4.5 AC, ab
Deep Lakes	27.6 ± 12.9 A, a	6.9 ± 8.2 B, b	17.3 ± 8.5 B, ab	18.6 ± 7 AB, ab
Streams	19.6 ± 3.4 A, a	5.4 ± 4.8 D, b	5.4 ± 7.2 C, b	3.7 ± 2.6 AC, b

*Note*: Shared uppercase letters in a single column indicate no difference between wetland types for each species and shared lowercase letters in a single row indicate no difference among species for each wetland type (Kruskal–Wallis *H* test, *p* < .05). Deep Lakes included Deep *Carex* and Deep Open Lakes that were differentiated in later but not earlier years of the study.

**TABLE A6:** Macroinvertebrate biomass density (mean ± SE) in cores (mg C/m^2^ of benthic surface area) and net sweeps (mg C/m^3^ of water column within emergent vegetation), 2017–2018, in different wetland types.

Wetland type	Cores (mg C/m^2^)	Net sweeps (mg C/m^3^)
Shallow *Arctophila*	247.8 ± 53 (14) A	314.7 ± 99 (12) ABCD
Deep *Arctophila*	203.3 ± 56.1 (10) A	160.6 ± 39.4 (10) A
Shallow *Carex*	473.3 ± 112.4 (11) BC	449.8 ± 94.4 (11) B
Deep Lakes	181.2 ± 57.9 (11) A	211.6 ± 53.0 (11) C
Deep *Carex*	173.5 ± 44.5 (7) A	286.8 ± 62.7 (7) BCD
Deep Open Lakes	194.7 ± 154 (4) A	80.1 ± 53.7 (4) AD
Streams	421.8 ± 324.2 (6) AC	165.3 ± 56.7 (6) AC

*Note*: Sample sizes are in parentheses. Values with shared letters in each column do not differ (Kruskal–Wallis *H* test, *p* > .05). Estimates include all invertebrate taxa retained by a 0.5‐mm sieve (excluding meiofaunal Oligochaeta. Deep *Carex* and Deep Open Lakes were not distinguished in earlier years of the study and were combined as Deep Lakes in most analyses.

**TABLE A7:** Relative prevalence of specific behaviors within broader categories.

	Observations with specific behaviors (%)				Proportion of broader behavior group (mean % ± SD)			
	LTDU	STEI	SPEI	KIEI	LTDU	STEI	SPEI	KIEI
Loafing behaviors	78.5	76.1	73.6	57.4				
Alert	13.5	26.6	27.3	13.1	8.1 ± 22.9	18.5 ± 34.4	22.5 ± 38.2	13.5 ± 31.1
Scanning	0.2	3.3	1.1	5.2	0 ± 1.1	2.8 ± 15.2	1.5 ± 12.2	6 ± 21.8
Preening	14.4	26.9	21.6	26.8	10.3 ± 26.2	20.7 ± 35	14.6 ± 31	27.2 ± 39.4
Bathing	1	3.9	2.3	1.3	0.4 ± 4.7	1.3 ± 7.9	0.3 ± 1.8	0.7 ± 5.6
Resting	10.6	9.4	17	7.8	5.4 ± 18.5	3.6 ± 14.6	9.4 ± 24.2	5.7 ± 18.6
Sleeping	64.9	36.6	42	30.1	71.4 ± 44.4	37.9 ± 47.1	48.4 ± 47.1	43.4 ± 46.7
Undefined loafing	5.1	13.9	4.5	2.6	4.5 ± 20	15.2 ± 35.8	3.2 ± 14.5	3.4 ± 18.1
Foraging behaviors	35.1	50.3	48.4	59.4				
Diving	26.9	3	NA	0.7	64.1 ± 46.2	5 ± 21.4	NA	0.2 ± 2.1
Tipping up/Dabbling	1.1	13.6	14.8	16.3	2.1 ± 14.2	16.6 ± 34.1	20.9 ± 36.4	12.8 ± 26.5
Head dipping	10.7	42.9	43.2	54.2	21.2 ± 38.6	67 ± 43.6	74 ± 40	81 ± 32.8
Skimming	0.8	1.8	2.3	3.3	2 ± 14.1	1 ± 6.5	0.4 ± 1.8	3.6 ± 18
Emergent grazing	NA	2.1	NA	0.7	NA	2.4 ± 14	NA	0.1 ± 1.3
Upland grazing	NA	0.6	1.1	NA	NA	0.1 ± 1.1	2.3 ± 15.1	NA
Undefined foraging	4.2	5.4	2.3	2	10.6 ± 30.8	7.8 ± 25.9	2.4 ± 15.1	2.3 ± 14.7
Movement behaviors	24.1	30.9	44	33.5				
Swimming	3.4	3	1.1	2.6	89.3 ± 25.2	82.9 ± 30.2	70.4 ± 39.3	76.6 ± 37
Walking	25.8	32.6	37.5	28.8	0.3 ± 2.2	9.2 ± 24	20 ± 34.1	14.3 ± 28.7
Flying	2.7	4.2	5.7	2	3.8 ± 13.8	4.3 ± 14.4	7 ± 19.7	3 ± 14.9
Undefined movement	0.6	5.7	17	9.8	6.6 ± 21.8	3.6 ± 14	2.5 ± 15.8	6 ± 23.5
Reproductive behaviors	3.7	12.5	9.9	12.9				
Courtship	3.4	11.8	9.1	10.5	76.9 ± 40.6	76.1 ± 39.5	88.9 ± 33.3	75.8 ± 41
Enticing	NA	0.3	1.1	NA	NA	1.1 ± 7.3	5.6 ± 16.7	NA
Copulation	0.3	0.3	1.1	1.3	5.8 ± 21.6	0.7 ± 4.9	5.6 ± 16.7	10 ± 30.8
Nesting	NA	0.3	NA	NA	NA	2.1 ± 14.6	NA	NA
Aggression	0.6	3.3	NA	2.6	13.5 ± 33.3	19.5 ± 37.3	NA	14.2 ± 32.1
Undefined reproductive behavior	0.2	0.3	NA	NA	3.8 ± 19.6	0.5 ± 3.6	NA	NA

*Note*: Species and sample sizes are long‐tailed duck (LTDU, 624 observations), Steller's eider (STEI, 331), spectacled eider (SPEI, 88), and king eider (KIEI, 153). Values in the left section are the proportions of total behavior observations (frequency of occurrence) during which a given behavior was observed. Values in the right section are mean percentages ± SD of each broader behavior category (loafing, foraging, movement, and reproduction). Some behaviors were not observed in a given species, indicated by NA. Diving recorded here only applies to behaviors recorded at 20‐s intervals, not continuous diving behavior (Table A8).

**TABLE A8:** Frequency and duration of dives by female sea ducks, 2017–2019.

Year	Variable	Long‐tailed duck	Steller's eider	Spectacled eider	King eider
2017	5‐min focal periods (*n*)	156	127	65	81
	Focal periods with dives (%)	26.8	4.7	1.5	1.2
	Mean dives/period ± SD	2.35 ± 4.66	0.64 ± 3.33	0.02 ± 0.12	0.02 ± 0.22
	Dive duration ± SD (s)	16 ± 9.8	10.4 ± 5.7	–	–
2018	5‐min focal periods (*n*)	468	204	20	40
	Focal periods with dives (%)	17.3	1.5	0	0
	Mean dives/period	1.92 ± 5.23	0.32 ± 2.70	0	0
	Dive duration ± SD (s)	12.3 ± 7	7 ± 2.5	–	–
2019	5‐min focal periods (*n*)	–	–	3	34
	Focal periods with dives (%)	–	–	0	0
	Mean dives/period	–	–	0	0
	Dive duration ± SD (s)	–	–	–	–
All	5‐min focal periods (*n*)	624	331	88	155
	Focal periods with dives (%)	19.7	2.7	1	0.7
	Mean dives/period	2.09 ± 5.14	0.44 ± 2.94	0.01 ± 0.11	0.01 ± 0.16
	Dive duration ± SD (s)	13.6 ± 8.1	8.9 ± 4.9	1 ± 0	9 ± 1.4

*Note*: Sampling effort in 2019 emphasized spectacled and king eiders only. Note that very few dives were recorded for spectacled and king eiders.

## References

[ece310375-bib-0001] Alisauskas, R. T. , & Devink, J.‐M. (2015). Breeding costs, nutrient reserves, and cross‐seasonal effects: Dealing with deficits in sea ducks. In J.‐P. L. Ducks, Savard , D. V. Derksen , D. Esler , & J. M. Eadie (Eds.), Ecology and conservation of North American Sea (pp. 125–168). CRC Press.

[ece310375-bib-0002] Amundson, C. L. , Flint, P. L. , Stehn, R. A. , Platte, R. M. , Wilson, H. M. , Larned, W. W. , & Fischer, J. B. (2019). Spatio‐temporal population change of Arctic‐breeding waterbirds on the Arctic coastal plain of Alaska. Avian Conservation and Ecology, 14, 18.

[ece310375-bib-0003] Andresen, C. G. , Lara, M. J. , Tweedie, C. E. , & Lougheed, V. L. (2017). Rising plant‐mediated methane emissions from arctic wetlands. Global Change Biology, 23, 1128–1139. 10.1111/gcb.13469 27541438

[ece310375-bib-0004] Andresen, C. G. , & Lougheed, V. L. (2015). Disappearing Arctic tundra ponds: Fine‐scale analysis of surface hydrology in drained thaw lake basins over a 65 year period (1948–2013). Journal of Geophysical Research: Biogeosciences, 120, 466–479.

[ece310375-bib-0005] Bart, J. , & Earnst, S. L. (2005). Breeding ecology of spectacled eiders *Somateria fischeri* in northern Alaska. Wild, 55, 85–100.

[ece310375-bib-0006] Bergman, R. D. , Howard, R. L. , Abraham, K. F. , & Weller, M. W. (1977). Water birds and their wetland resources in relation to oil development at Storkerson Point, Alaska . U.S. Fish Wild. Serv. Resource Publication 129, Washington, DC, USA. 38 pp.

[ece310375-bib-0007] BirdLife International . (2022a). Species factsheet: *Clangula hyemalis* . http://datazone.birdlife.org/species/factsheet/long‐tailed‐duck‐clangula‐hyemalis

[ece310375-bib-0008] BirdLife International . (2022b). Species factsheet: *Polysticta stelleri* . http://datazone.birdlife.org/species/factsheet/stellers‐eider‐polysticta‐stelleri

[ece310375-bib-0009] BirdLife International . (2022c). Species factsheet: *Somateria fischeri* . http://datazone.birdlife.org/species/factsheet/spectacled‐eider‐somateria‐fischeri

[ece310375-bib-0010] BirdLife International . (2022d). Species factsheet: *Somateria spectabilis* . http://datazone.birdlife.org/species/factsheet/king‐eider‐somateria‐spectabilis

[ece310375-bib-0011] Bond, J. C. , Esler, D. , & Hobson, K. A. (2007). Isotopic evidence for sources of nutrients allocated to clutch formation by harlequin ducks. Condor, 109, 698–704.

[ece310375-bib-0012] Bowman, T. D. , Silverman, E. D. , Gililand, S. G. , & Leiness, J. B. (2015). Status and trends of North American sea ducks. In J.‐P. L. Savard , D. V. Derksen , D. Esler , & J. M. Eadie (Eds.), Ecology and conservation of North American Sea Ducks. Studies in avian biology (no. 46). (pp. 1–28). CRC Press.

[ece310375-bib-0013] Butler, M. , Miller, M. C. , & Mozley, S. (1980). Macrobenthos. In A. Barrow & J. E. Hobbie (Eds.), Limnology of tundra ponds (pp. 297–339). Downden, Hutchinson, and Ross.

[ece310375-bib-0014] Clarke, K. R. , & Gorley, R. N. (2015). PRIMER v7: User manual/tutorial. PRIMER‐e (Quest Research Limited).

[ece310375-bib-0015] Coulson, J. C. (2010). A long‐term study of the population dynamics of common eiders *Somateria mollissima*: Why do several parameters fluctuate markedly? Bird Study, 57, 1–18.

[ece310375-bib-0016] Cowardin, L. M. , Carter, V. , Golet, F. C. , & LaRoe, E. T. (1979). Classification of wetlands and deepwater habitats of the United States . U.S. Fish Wild. Serv. report. Washington, D.C. 103 pp.

[ece310375-bib-0017] Cox, C. J. , Stone, R. S. , Douglas, D. C. , Stanitski, D. M. , Divoky, G. J. , Dutton, G. S. , Sweeney, C. , George, J. C. , & Longenecker, D. U. (2017). Drivers and environmental responses to the changing annual snow cycle of northern Alaska. Bulletin of the American Meteorological Society, 98, 2559–2577.

[ece310375-bib-0018] Derksen, D. V. , Rothe, T. C. , & Eldridge, W. D. (1981). Use of wetland habitats by birds in the National Petroleum Reserve – Alaska . U.S. Fish Wildl. Serv. Resour. Publ. 141. 27 pp.

[ece310375-bib-0019] Dunham, K. D. , Osnas, E. E. , Frost, C. J. , Fischer, J. B. , & Grand, J. B. (2021). Assessing recovery of spectacled eiders using a Bayesian decision analysis. PLoS One, 16(7), e0253895. 34197512 10.1371/journal.pone.0253895 PMC8248636

[ece310375-bib-0020] Graff, N. (2021). Breeding ecology of Steller's and spectacled eiders nesting near Utqiaġvik, Alaska, 2018–2019 . U.S. Fish Wildl. Serv., Fairbanks Fish Wildl. Field Office, Fairbanks, Alaska, USA.

[ece310375-bib-0021] Higgins, R. P. , & Thiel, H. (1988). Introduction to the study of meiofauna. Smithsonian Institution Press.

[ece310375-bib-0022] Hollander, M. , Wolfe, D. A. , & Chicken, E. (2015). Nonparametric statistical methods (3rd ed.). Wiley.

[ece310375-bib-0023] Hummon, W. D. (1981). Extraction by sieving: A biased procedure in studies of stream meiobenthos. Transactions of the American Microscopical Society, 100, 278–284.

[ece310375-bib-0024] Jónasson, P. M. (1955). The efficiency of sieving techniques for sampling freshwater bottom fauna. Oikos, 6, 183–207.

[ece310375-bib-0025] Kondratiev, A. V. , & Zadorina, L. V. (1992). Comparative ecology of king eider *Somateria spectabilis* and spectacled eider *S. fischeri* in Chaun tundra. Zoologichesky Zhurnal, 71, 99–108. [in Russian].

[ece310375-bib-0026] Kondratyev, A. V. (1999). Foraging strategies and habitat use of sea ducks breeding in northeast Russia. In R. I. Goudie , M. R. Petersen , & G. J. Robertson (Eds.), Behaviour and ecology of sea ducks (pp. 52–59). Can. Wildl. Serv. Occas. Paper 100.

[ece310375-bib-0027] Lara, M. J. , Johnson, D. R. , Andresen, C. , Hollister, R. D. , & Tweedie, C. E. (2016). Peak season carbon exchange shifts from a sink to a source following 50+ years of herbivore exclusion in an Arctic tundra ecosystem. Journal of Ecology, 105, 122–131. 10.1111/1365-2745.12654

[ece310375-bib-0028] Lougheed, V. L. , Butler, M. G. , McEwen, D. C. , & Hobbie, J. E. (2011). Changes in tundra pond limnology: re‐sampling Alaskan ponds after 40 years. Ambio, 40, 589–599. 21954722 10.1007/s13280-011-0165-1 PMC3357870

[ece310375-bib-0029] Lovvorn, J. R. (1989). Food defendability and antipredator tactics: Implications for dominance and pairing in canvasbacks. Condor, 91, 826–836.

[ece310375-bib-0030] Lovvorn, J. R. , De La Cruz, S. E. W. , Takekawa, J. Y. , Shaskey, L. E. , & Richman, S. E. (2013). Niche overlap, threshold food densities, and limits to prey depletion for a diving duck assemblage in an estuarine bay. Marine Ecology Progress Series, 476, 251–268.

[ece310375-bib-0031] Marsh, P. , Russell, M. , Pohl, S. , Haywood, H. , & Onclin, C. (2009). Changes in thaw lake drainage in the Western Canadian Arctic from 1950–2000. Hydrological Processes, 23, 145–158.

[ece310375-bib-0032] McEwen, D. C. , & Butler, M. G. (2018). Growing‐season temperature change across four decades in an Arctic tundra pond. Arctic, 71, 281–291.

[ece310375-bib-0033] Miller, M. W. C. , Lovvorn, J. R. , Graff, N. R. , & Stellrecht, N. C. (2022). Use of marine vs. freshwater proteins for egg‐laying and incubation by sea ducks breeding in Arctic tundra. Ecosphere, 13, e4138.

[ece310375-bib-0034] Nalepa, T. F. , & Robertson, A. (1981). Screen mesh size affects estimates of macro‐ and meio‐benthos abundance and biomass in the Great Lakes. Canadian Journal of Fisheries and Aquatic Sciences, 38, 1027–1034.

[ece310375-bib-0035] Oppel, S. , Powell, A. N. , & Butler, M. G. (2011). King eider foraging during the pre‐breeding period in Alaska. Condor, 113, 52–60.

[ece310375-bib-0036] Plesh, S. P. , Lovvorn, J. R. , & Miller, M. W. C. (2023). Organic matter sources and flows in tundra wetland food webs. PLos One, 18(5), e0286368. 37235582 10.1371/journal.pone.0286368 PMC10218757

[ece310375-bib-0037] Previdi, M. , Smith, K. L. , & Polvani, L. M. (2021). Arctic amplification of climate change: A review of underlying mechanisms. Environmental Research Letters, 16, 093003.

[ece310375-bib-0038] Quakenbush, L. , Suydam, R. , Obritcshkewitsch, T. , & Deering, M. (2004). Breeding biology of Steller's eiders (*Polysticta stelleri*) near Barrow, Alaska, 1991–99. Arctic, 57, 166–182.

[ece310375-bib-0039] R Core Team . (2021). R: A language and environment for statistical computing. R Foundation for Statistical Computing. https://www.R‐project.org/

[ece310375-bib-0052] Rantanen, M. , Karpechko, A. Y. , Lipponen, A. , Nordling, K. , Hyvärinen, O. , Ruosteenoja, K. , Vihma, T. , & Laaksonen, A. (2022). The Arctic has warmed nearly four times faster than the globe since 1979. Communications Earth & Environment, 3(1), 168. 10.1038/s43247-022-00498-3

[ece310375-bib-0040] Raynolds, M. K. , & Walker, D. A. (2016). Increased wetness confounds Landsat‐derived NDVI trends in the central Alaska North Slope region, 1985–2011. Environmental Research Letters, 11, 085004.

[ece310375-bib-0041] Reyes, F. R. , & Lougheed, V. L. (2015). Rapid nutrient release from permafrost thaw in Arctic aquatic ecosystems. Arctic, Antarctic, and Alpine Research, 47, 35–48.

[ece310375-bib-0042] Safine, D. E. (2013). Breeding ecology of Steller's and spectacled eiders nesting near Barrow, Alaska, 2012 . U.S. Fish and Wildlife Service, Fairbanks, Alaska, USA. Technical report. 56 pp.

[ece310375-bib-0043] Smith, L. C. , Sheng, Y. , MacDonald, G. G. , & Hinzman, L. D. (2005). Disappearing Arctic lakes. Science, 308, 1429. 15933192 10.1126/science.1108142

[ece310375-bib-0044] Smith, P. A. , McKinnon, L. , Meltofte, H. , Lanctot, R. B. , Fox, A. D. , Leafloor, J. O. , Soloviev, M. , Franke, A. , Falk, K. , Golovatin, M. , Sokolov, V. , Sokolov, A. , & Smith, A. C. (2020). Status and trends of tundra birds across the circumpolar Arctic. Ambio, 49, 732–748. 31955397 10.1007/s13280-019-01308-5 PMC6989588

[ece310375-bib-0045] Smol, J. P. , & Douglas, M. S. V. (2007). Crossing the final ecological threshold in high Arctic ponds. Proceedings of the National Academy of Sciences of the United States of America, 104, 12395–12397. 17606917 10.1073/pnas.0702777104 PMC1941480

[ece310375-bib-0046] Solovieva, D. V. (2005). Time budgets and foraging ecology of the Steller's eider *Polysticta stelleri* . Avian Ecology and Behaviour: Proceedings of the Biological Station “Rybachy”, 13, 11–24.

[ece310375-bib-0047] Suydam, R. S. , Dickson, D. L. , Fadely, J. B. , & Quakenbush, L. T. (2000). Population declines of king and common eiders of the Beaufort Sea. Condor, 102, 219–222.

[ece310375-bib-0048] U.S. Fish and Wildlife Service . (2019). Status assessment of the Alaska‐breeding population of Steller's eiders . U.S. Fish Wildl. Serv. report. Fairbanks, Alaska, USA. 149 pp.

[ece310375-bib-0049] U.S. Fish and Wildlife Service . (2021). Species status assessment for the spectacled eider . U.S. Fish Wildl. Serv. report. Fairbanks, Alaska, USA. 150 pp.

[ece310375-bib-0050] U.S. Geologic Survey . (2021). Interferometric Synthetic Aperture Radar (IFSAR) Alaska . 10.5066/P9C064CO

[ece310375-bib-0051] Wauchope, H. S. , Shaw, J. D. , Varpe, Ø. , Lappo, E. G. , Boertmann, D. , Lanctot, R. B. , & Fuller, R. A. (2017). Rapid climate‐driven loss of breeding habitat for Arctic migratory birds. Global Change Biology, 23, 1085–1094. 27362976 10.1111/gcb.13404

